# Tormentic Acid Ameliorates Hepatic Fibrosis *in vivo* by Inhibiting Glycerophospholipids Metabolism and PI3K/Akt/mTOR and NF-κB Pathways: Based on Transcriptomics and Metabolomics

**DOI:** 10.3389/fphar.2022.801982

**Published:** 2022-03-11

**Authors:** Xing Lin, Yuanyuan Wei, Yan Li, Yuhua Xiong, Bin Fang, Cuiyu Li, Quanfang Huang, Renbin Huang, Jinbin Wei

**Affiliations:** ^1^ Guangxi Medical University Life Sciences Institute, Nanning, China; ^2^ Pharmaceutical College, Guangxi Medical University, Nanning, China; ^3^ The Pharmaceutical Department, Guangxi University of Chinese Medicine First Affiliated Hospital, Nanning, China

**Keywords:** liver fibrosis, tormentic acid, transcriptomics, metabolomics, glycerophospholipid metabolism pathway

## Abstract

This study aimed to investigate the effects and underlying mechanisms of tormentic acid (TA) on carbon tetrachloride (CCl_4_)-induced liver fibrosis in rats. The rats were intragastrically administered with 50% CCl_4_ for 9 weeks to induce hepatic fibrosis, followed by various agents for 6 weeks. Transcriptomic analysis was carried out to predict the potential targets, and then multiple examinations were performed to verify the prediction. The results showed that TA significantly alleviated liver injury and fibrosis, as evidenced by the ameliorative pathological tissue, low transaminase activity, and decreased collagen accumulation. Besides, TA markedly reduced hepatocyte apoptosis by regulating the expression of caspase-3 and Bcl-2 families. The transcriptomic analysis revealed 2,173 differentially expressed genes (DEGs) between the TA and model groups, which could be enriched in the metabolic pathways and the PI3K/Akt and NF-κB signaling pathways. The metabolomics analysis showed that TA could regulate the glycerophospholipid metabolism pathway by regulating the synthesis of phosphatidylserines, phosphatidylethanolamines and phosphatidylcholines. Moreover, the integrative analysis of the transcriptomics and metabolomics data indicated that TA inhibited the glycerophospholipid metabolism pathway by inhibiting the expression of LPCAT4, PTDSS2, PLA2G2A and CEPT1. In addition, the relevant signaling pathways analysis confirmed that TA inhibited HSCs activation by blocking the PI3K/Akt/mTOR pathway and ameliorated inflammatory injury by inhibiting the NF-κB pathway. In conclusion, TA significantly alleviates liver fibrosis *in vivo* by inhibiting the glycerophospholipid metabolism pathway and the PI3K/Akt/mTOR and NF-κB signaling pathways.

## Introduction

Liver fibrosis caused by various chronic stimuli, such as viruses, alcohol, and autoimmune diseases, is a reversible pathological process. Without appropriate intervention, it can progress to cirrhosis, portal hypertension, and hepatocellular carcinoma, leading to high morbidity and mortality (Parola and Pinzani, 2019). Thus, it is urgent to develop diagnostic techniques and new agents to prevent liver fibrosis progression.

Multi-omics analysis has been considered a useful tool to study the pathogenesis of various diseases and drug discovery because of its advantage in providing insight into the potential biomarkers and mechanism as a whole. Transcriptome analysis can detect the complete set of RNA transcripts, which is helpful to expose the molecular mechanisms of the anti-hepatic fibrosis drugs (Zhang et al., 2019). Metabolomics analysis can provide the global metabolic profile and identify the differently expressed metabolites. The integrative analysis of transcriptome and metabolome has been increasingly used to investigate the complicated mechanisms of liver diseases (Zhao et al., 2020; Tang et al., 2021).

Applying traditional herb medicines and their active ingredients may provide a comprehensive advantage in pathologically complicated liver fibrosis (Sun et al., 2019). *Potentilla chinensis* Ser*.* is a folk herb for treating immune disorders, which has been found to have a protective effect on liver injury (Li et al., 2004). In our previous experiments, we have extracted an ingredient from this herb and identified it as tormentic acid (TA). But whether TA has the hepatoprotective effects remains unclear so far. Thus, in this study, the potential target genes and the network of the relevant pathways were predicted by transcriptomic analysis. Then the predictions were verified by performing metabolomic analysis and detecting the relevant signaling pathways. This study aimed to elucidate the protective effects and the underlying mechanisms of TA against CCl_4_-induced liver fibrosis in rats, which tried to provide the theoretical basis for its application in the treatment of liver fibrosis in the future.

## Materials and Methods

### Animals and Treatment

Male Sprague–Dawley (SD) rats (SPF, 180 ± 10 g) were provided by the Experimental Animal Center of Guangxi Medical University (Guangxi, China). The animal experiment was approved by the Institutional Ethical Committee of Guangxi Medical University. The treatment was performed as shown in Supplemental Figure S1. Briefly, after 1 week of acclimation, sixty rats were randomly divided into six groups (10 rats/group): the normal control group, TA control group (3.0 mg/kg TA), model group, positive control group (0.2 mg/kg colchicine (Zhang et al., 2020)), and TA-treated groups (3.0 or 1.5 mg/kg TA). The rats were intragastrically administered with 2 ml/kg CCl_4_ (50% oil solution) twice a week for 9 weeks to induce liver fibrosis, followed by TA (3.0 or 1.5 mg/kg) or colchicine (0.2 mg/kg) for 6 weeks. At the end of the treatment, all animals were fasted for 12 h and then anesthetized by intraperitoneal injection of 3% sodium pentobarbital (1.0 ml/kg). Blood and liver samples were collected immediately.

### Histopathological Examination

Liver samples were fixed in 4% paraformaldehyde, embedded in paraffin and sectioned into 4 μm slices. Then the slices were stained with the hematoxylin-eosin (H and E) staining to observe the hepatic pathological changes. Sirius red staining was used to assess collagen deposition, and the semi-qualification for collagen area was analyzed using ImagePro Plus 6 (Media Cybernetics, Inc.). Moreover, hepatocyte apoptosis was evaluated by the TUNEL staining (Beyotime, Shanghai, China).

### Measurement for Hepatic Function Index

The activities of the serum enzymes, including alanine aminotransferase (ALT), aspartate aminotransferase (AST), albumin (ALB) and total bilirubin (TBIL), were estimated with an automatic biochemistry analyzer (Hitachi, Ltd., Kokubunji, Tokyo, Japan).

### Measurement for Collagen-Related Indicators

Hyaluronic acid (HA), laminin (LN), procollagen type Ⅲ (HPCⅢ) and collagen type IV (Col- IV) contents were tested by the enzyme-linked immunosorbent assay (ELISA) kits (Wuhan Cusabio Bioengineering Co. Ltd., Wuhan, China). The hepatic hydroxyproline (Hyp) content was determined using a commercially available kit (Nanjing Jiancheng Bio-engineering Institute, Nanjing, China).

### Detection of Oxidative Stress, Lipid Peroxidation Levels, and Inflammatory Cytokines

The activities of SOD, GSH-Px, GSH-Rd and MDA in the hepatic tissue were detected using commercially available kits (Nanjing Jiancheng Bio-engineering Institute, Nanjing, China). The contents of interleukin-6 (IL-6), interleukin-10 (IL-10) and tumor necrosis factor-α (TNF-α) were determined using the ELISA kits (Wuhan elabscience Biotechnology Co., Ltd., Wuhan, China) according to the assay procedure. The content of monocyte chemoattractant protein 1 (MCP-1) was detected using the ELISA kit (Sigma-Aldrich).

### Transcriptomic Analysis

Total RNA was extracted from liver tissues with Trizol reagent (Thermo Fisher Scientific). RNA sequences analysis was performed by Shanghai Jiayin Biotechnology Co., Ltd (Yu et al., 2018). Briefly, RNA-seq transcriptome library was created with NEBNext UltraTM RNA Library Prep Kit (NEB #E7490). mRNA was isolated according to polyA selection method by oligo (dT) beads (AMPure XP system) and then fragmented by fragmentation buffer. Next, double-stranded cDNA was synthesized using a SuperScript double-stranded cDNA synthesis kit (Invitrogen, CA) with random hexamer primers (Illumina). Libraries were selected for cDNA target fragments of 200–300 bp on 2% Low Range Ultra Agarose. After being quantified by TBS380, the paired-end RNA-seq library was sequenced with the Illumina Novaseq 6000 PE150 (Illumina). Sequence readers were aligned with Trimmomaticfor quality control (Bolger et al., 2014), and then adapter sequences and poor quality reads were removed using Cutadapt (Chen et al., 2014). Quality-filtered reads were then mapped to the human genome (hg19) using STAR. Read counts were calculated with HTSeq-count. mRNA level was quantified by the value of fragments per kilobase of exon per million mapped reads (FPKM). Differentially-expressed mRNAs were identified as those with *p*-values<0.05 and | log2 (fold change) |>1 using R package DESeq2.

### Metabolomic Analysis

Metabolomics was analyzed using Ultra-performance liquid chromatography equipped with quadrupole time-of-flight mass spectroscopy (UPLC-Xevo G2-XS QTof, Waters, United States) as previously described (Zhao et al., 2020). Approximately 50 mg liver tissue was homogenized with 500 μl lysis buffer (acetonitrile: methanol: extra-pure water = 2:2:1) at 4°C for 20 s and centrifuged at 12,000 g for 10 min at 4°C. The supernatants were filtered through 0.22 μm disc filters, and 200 μl was used for metabolites analysis. Quality control (QC) samples were prepared by pooling and aliquoting sample extracts. The distribution of QC data in principal component analysis (PCA) and the relative standard deviations (RSDs) of retention times was used to evaluate data quality.

Mass spectrometry condition was: ES^+^, capillary: 250 kV, source temperature: 150°C, desolvation temperature: 350°C, acquisition times: 15 min, acquisition mode: positive, low mass: 50 Da, high mass: 1,000 Da, scan time: 0.5 s, collision energy: 6 V, ramp collision energy 10–20 V, cone voltage: 40 V. LockSpray properties: “Acquire LockSpray-Do not apply correction.” Leucine enkephalin was used for LockSpray. Nitrogen was used as both cone gas (50 l/h) and desolvation gas (800 l/h). Argon was set as collision gas. The liquid chromatography conditions were: Acquity UPLC^®^BEH C18 column (50 × 2.1 mm, 1.7 μm, Waters Crop.). The gradient elution was performed using HPLC-grade water and acetonitrile containing 0.1% formic acid (Supplemental Table S1). The data were analyzed using MassLynx V4.1, Progenesis QI V2.4 and EZinfo V3.0 (Waters Inc.). The metabolic pathways were analyzed with MetaboAnalyst 5.0.

### Real-Time Quantitative PCR Assay

The total RNA from liver tissues was extracted using the AxyPrep Total RNA Mini Preparation Kit (Corning Life Sciences Co., Ltd., Jiangsu, China). The RNA was reverse-transcribed using an RT-PCR kit (Takara Biotechnology, Dalian, China) to obtain cDNA. The mRNA expression was performed using the Applied 7,300 Fast Real-Time PCR detection system and SYBR Green I (Takara Biotechnology, Dalian, China) according to the manufacturer’s instructions. The expression of Col-I, Col-Ⅲ, TGF-β, Bax, Bcl-2, PI3K, Akt, P70S6k, mTOR, and NF-κB p65 were analyzed. GAPDH was used as an internal control. The primer sequences used in this study are shown in Supplemental Table S2. The result was analyzed using the 2^−ΔΔCt^ method.

### Western Blotting Assay

The proteins of liver samples were extracted with the RIPA buffer (Solarbio, Beijing, China), followed by centrifuging at 12,000 g for 15 min. Subsequently, the concentration of each protein sample was detected using the BCA Protein Assay Kit (Beyotime, Jiangsu, China). Finally, the protein sample was separately mixed with loading buffer and denatured in boiling water for 5 min.

The electrophoretic separation of the prepared proteins was performed on the sodium dodecyl sulfate-polyacrylamide gels (SDS-PAGE) and transfered to polyvinylidene fluoride (PVDF) membranes (Millipore, United States). The membranes were incubated with the primary antibodies overnight at 4°C: Akt, p-Akt, PI3K, p-PI3K, FAK, p-FAK, mTOR, p-mTOR, NF-κB p65, p-NF-κB p65 (1:1,000, Cell Signaling Technology Inc., Beverly, MA), IκBα, p-IκBα, IKKα/β, p-IKKα/β, Bcl-2, Bax, Caspase-3, and GAPDH (1:1,000, Proteintech, Chicago, IL), *α*-SMA, Col-I and Col-Ⅲ (1:500, Proteintech, Chicago, IL). The membranes were washed three times with TBST buffer, followed by incubation in the dark with fluorescence-labeled rabbit/mouse anti-goat IgG (Licor, United States) at 1:10,000 dilution under agitation for 1 h at room temperature. Finally, the membranes were scanned and quantified by Image Studio Lite software (LI-COR Biosciences, NE, United States).

### Statistical Analysis

The data were expressed as the means ± standard deviation. The statistical analyses were performed using SPSS 13.0 software (Chicago, IL). The one-way ANOVA was used to determine statistical differences between the groups. A *p*-value < 0.05 was considered statistically significant.

## Results

### TA Alleviated CCl_4_-Induced Liver Injury

**FIGURE 1:**
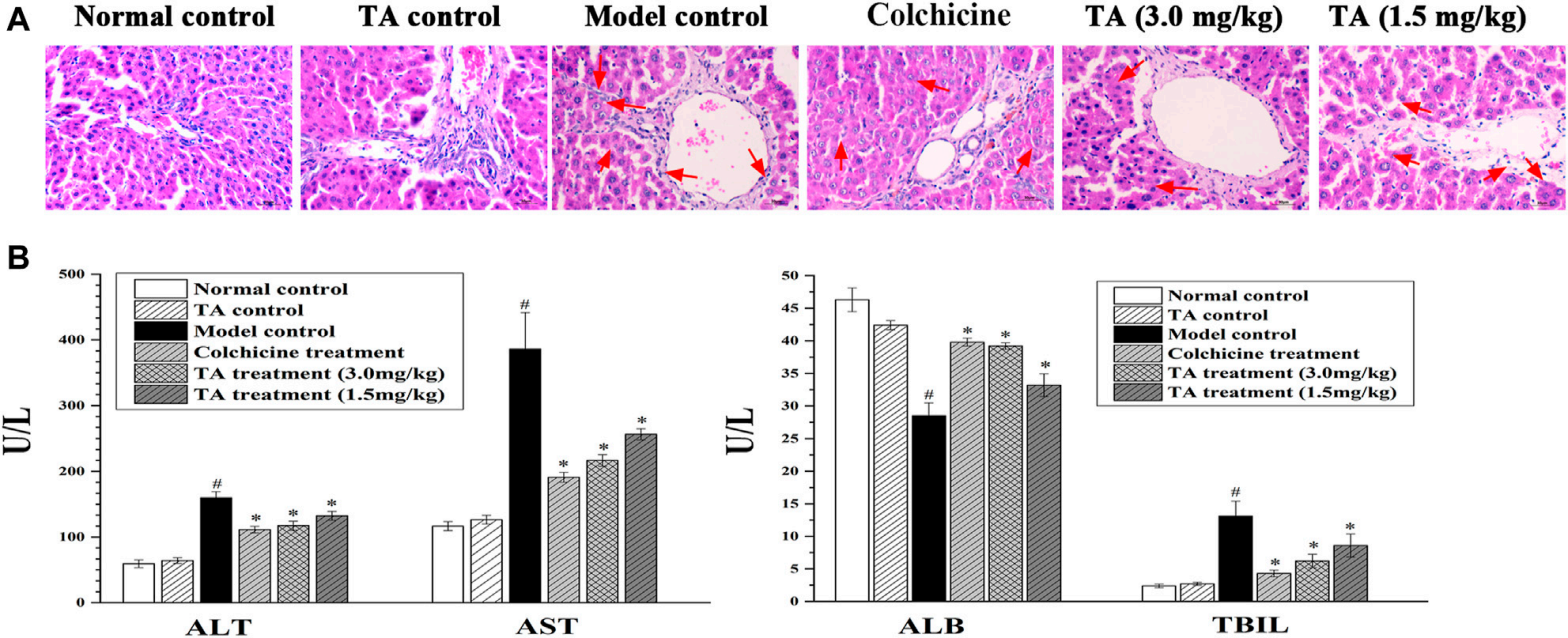
TA alleviated CCl4-induced liver injury. **(A)** Hepatic histological changes were observed by H and E staining (400 ×); the arrow indicates swelling or necrosis. **(B)** The activity of serum ALT, AST, ALB, and TBIL was measured using an automatic biochemistry analyzer. ^*^ *p* < 0.05 vs. the model control group. ^#^ *p* < 0.05 vs the normal control group.

Liver tissue pathological change was observed by H&E staining. As shown in Figure 1A, the liver tissues in the normal control and the TA control groups showed clear and intact lobules. However, in the model group, the liver lobules were destroyed, and the arrangement of liver cells was disordered. Treatment with colchicine or TA significantly ameliorated liver damage caused by CCl_4_, as evidenced by less necrosis.

Serum ALT, AST, TBIL, and ALB are the indicators of hepatic function. In this study, CCl_4_ exposure caused a significant increase in ALT, AST, and TBIL, while a decrease in ALB (Figure 1B); colchicine or TA treatment markedly reversed these abnormal changes, suggesting that TA could improve hepatic function.

### TA Reduced Collagen Accumulation

**FIGURE 2:**
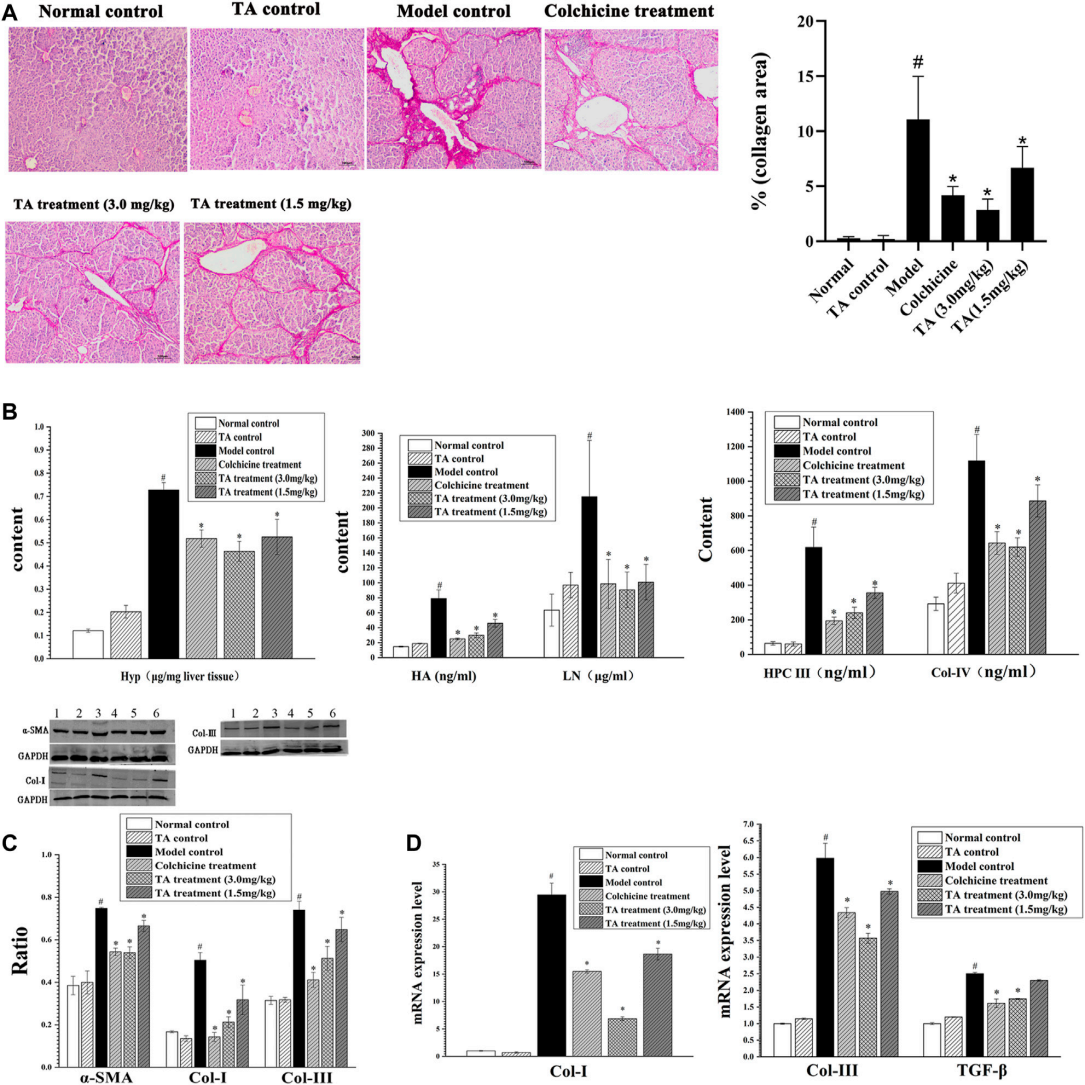
TA reduced collagen accumulation. **(A)** Collagen deposition in liver tissue was observed by Sirius staining (100×). **(B)** Effects of TA on Hyp, HA, LN, HPC Ⅲ and Col- IV were tested by commercially available kits. **(C)** The protein expression of *α*-SMA, Col-I and Col- III was detected by Western blot. **(D)** The mRNA expression of Col-I, Col-III and TGF-*β* was detected by qPCR analysis. Band one to six represented the normal control group, the TA control group, the model control group, the colchicine treatment group and the TA treatment groups (3.0 and 1.5 mg/kg), respectively. ^*^ *p* < 0.05 vs the model control group. ^#^ *p* < 0.05 vs the normal control group.

The feature of liver fibrosis is the excessive deposition of collagen fibers. The Sirius staining showed that the liver tissue sections from the normal control and TA control groups displayed little collagen deposition; conversely, mass connective tissue, continuous fibrotic septa, regenerated and alternated nodules were observed in the model control group (Figure 2A). Interestingly, treatment with TA significantly reduced collagen distribution and deposition. The same trend was found in the collagen area: CCl_4_ exposure caused a large-sized collagen area and TA treatment significantly decreased the size of the collagen area (Figure 2A). These data suggested that TA could inhibit fibrogenesis.

Hyp, HA, LN, HPC Ⅲ and Col- IV are the important biomarkers of hepatic fibrosis (Bo et al., 2020). As shown in Figure 2B, the content of these indicators in the model control group was significantly increased compared to the normal control group (*p* < *0.05*). However, the contents of these collagen-related biomarkers in the colchicine and TA-treated groups were decreased compared to the model group (*p* < *0.05*). Also, the Western blot and qPCR analysis showed that TA markedly inhibited the expression of Col-I and Col-III (Figures 2C,D).

α-SMA is an indicator of HSCs activation, and TGF-β is a cytokine to induce HSCs activation and proliferation. As shown in Figures 2C,D, CCl_4_ administration caused a significant increase in the expression of *α*-SMA and TGF-β; however, TA treatment significantly decreased the expression of *α*-SMA and TGF-*β*, suggesting that TA could inhibit HSCs activation, reducing collagen production.

### TA Decreased CCl_4_-Induced Hepatocyte Apoptosis

**FIGURE 3:**
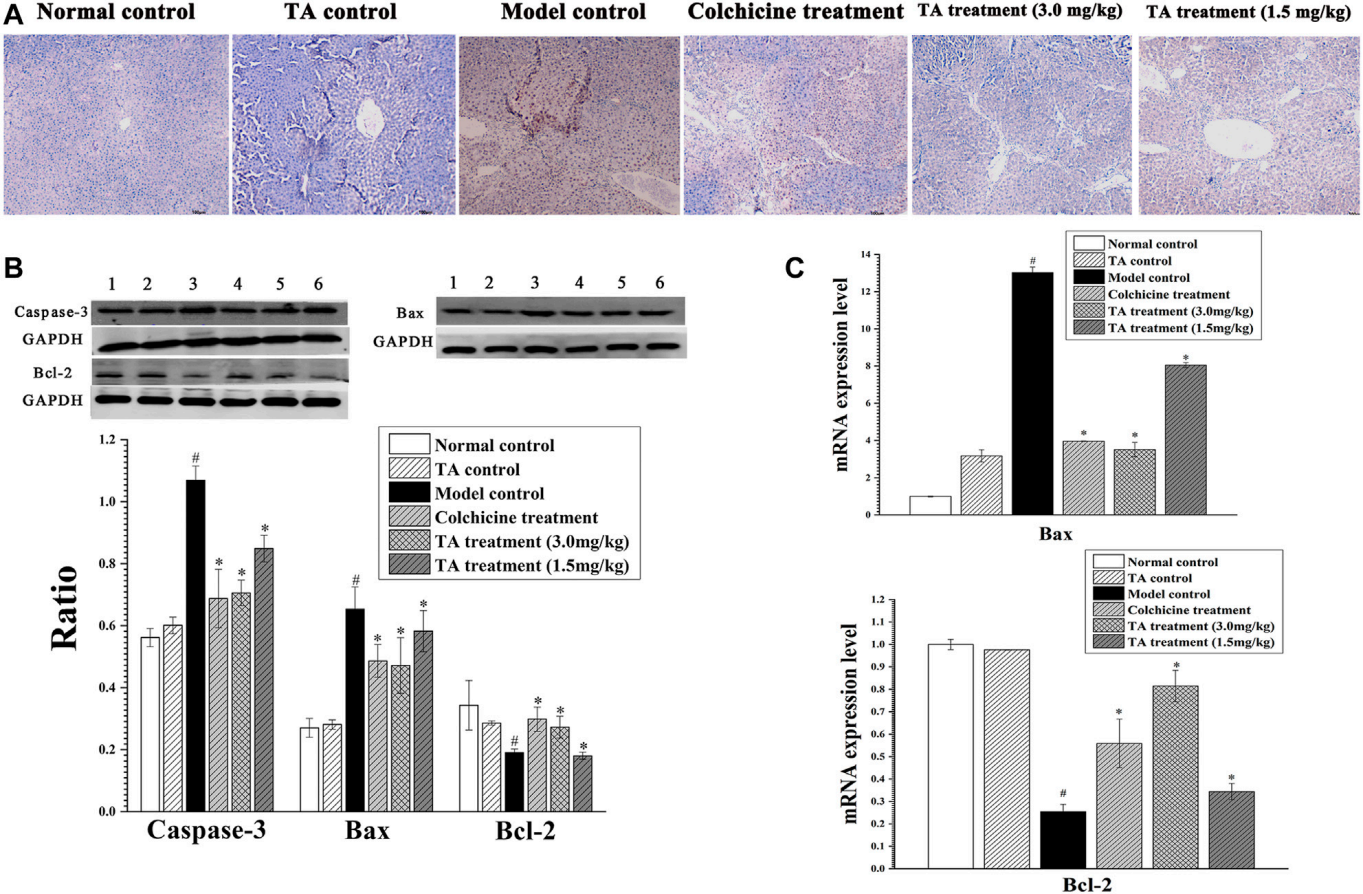
TA decreased hepatocyte apoptosis. **(A)** Hepatic apoptosis was observed by TUNEL staining (100×). **(B)** Effects of TA on the protein expression of Caspase-3, Bax and Bcl-2 in the liver tissue were detected by Western blot analysis. Band one to six represented the normal control group, the TA control group, the model control group, the colchicine treatment group and the TA treatment groups (3.0 and 1.5 mg/kg), respectively. **(C)** The mRNA expression of Bax and Bcl-2 was analyzed using qPCR analysis. ^*^ *p* < 0.05 vs the model control group. ^#^ *p* < 0.05 vs the normal control group.

The TUNEL staining revealed abundant brown spots, indicating CCl_4_ initiated serious hepatocytes apoptosis. In contrast, TA treatment markedly decreased apoptosis (Figure 3A). In addition, the apoptosis-related proteins Bax, Bcl-2, and caspase-3 were detected by Western blotting and qPCR. CCl_4_ exposure caused a significant increase in caspase-3 and Bax and a remarkable decrease in Bcl-2, whereas TA treatment markedly reversed these abnormal changes. These results suggested that TA could decrease CCl_4_-induced hepatocyte apoptosis (Figures 3B,C).

### TA Alleviated Oxidant Stress and Inflammation

**FIGURE 4:**
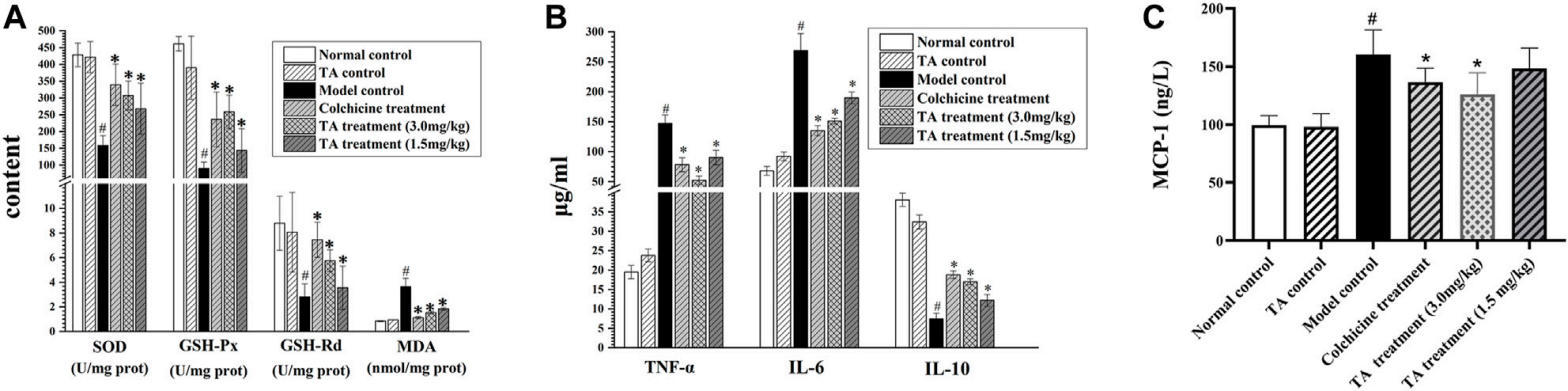
TA alleviated oxidative stress and inflammation. **(A)** Effects of TA on the content of MDA, SOD, GSH-Rd and GSH-Px in liver tissue. **(B)** The content of TNF-α, Il-6 and IL-10 in liver tissue. **(C)** The content of MCP-1 was detected using an ELISA kit. ^*^ *p* < 0.05 vs the model control group. ^#^ *p* < 0.05 vs. the normal control group.

As shown in Figure 4A, CCl_4_ exposure remarkably decreased the activity of SOD, GSH-Px and GSH-Rd, but increased MDA activity; however, TA treatment significantly reversed these abnormal changes, suggesting that TA could alleviate oxidant stress and lipid peroxidation by restoring the activity of antioxidant enzymes.

Besides, the inflammatory cytokines, including TNF-α, IL-6 and IL-10, were detected. As shown in Figure 4B, compared with the normal control group, the levels of IL-6 and TNF-α in the liver tissues of the model control group were significantly increased (*p* < *0.05*), and the content of IL-10 was lessened; however, treatment with TA could reverse these abnormal expressions induced by CCl_4_. Monocyte chemoattractant protein 1 (MCP-1) is a marker of hepatic inflammation (Marsillach et al., 2005). The ELISA result showed that TA (3.0 mg/kg) could significantly reduce MCP-1 content (Figure 4C). These data indicated that TA was able to ease the CCl_4_-induced inflammatory response.

### TA Restored CCl_4_-Induced Transcriptomic Alterations

Transcriptomics analysis was conducted by RNA-seq as previously described (Yu et al., 2018). In this study, Q20 or Q30 in all the samples was more than 95% and the GC content was less than 50% (Supplemental Table S3). Moreover, most of the quality scores of the bases from all the samples were more than 20 (Supplementary Figure S2), and the sequence contents of A, T, C and G were nearly equal (Supplementary Figure S3). Besides, the uniquely mapped reads (%) from all the samples were more than 85% (Supplemental Table S4). The quality-control evaluations above confirmed that the RNA-seq method with high sequencing precision could be used in the following analysis.

**FIGURE 5:**
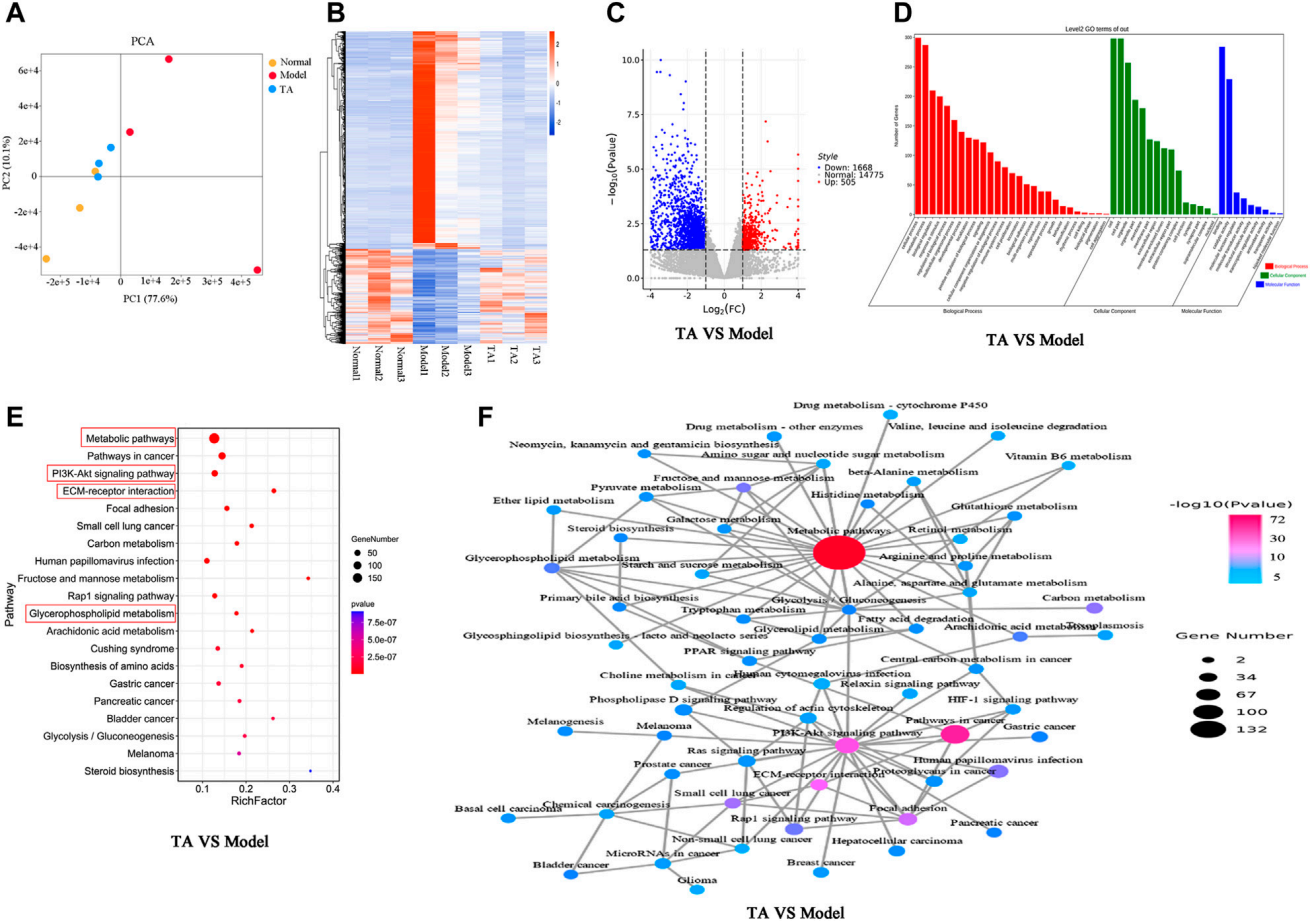
Transcriptomics analysis. **(A)**: PCA (Principal Component Analysis) diagram; **(B)**: Heatmap; **(C)**: Volcano plot; **(D)**: Gene ontology (GO) analysis; **(E)**: KEGG pathway analysis; **(F)**: Network of the relevant pathways.

The PCA (Principal Component Analysis) plots revealed a significant difference among the normal, model and TA groups (Figure 5A), which was further confirmed by the Heatmap plots (Figure 5B). Interestingly, both the plots indicated that CCl_4_ administration caused a remarkable change compared with the normal control, and TA treatment could restore CCl_4_-induced the abnormal expression of genes nearly to the normal level. For the model and normal groups (Model VS. Normal), there were 3,607 differentially expressed genes (DEGs) between them (Supplemental Fig. S 4 A). The Gene ontology (GO) analysis indicated that the DEGs were mainly involved in cell proliferation, immune system process, biological process and metabolites; meanwhile, the KEGG analysis showed that the DEGs were clustered in the metabolism-related pathways, ECM formation, PI3K/Akt signaling pathway, et al. (Supplementary Figures S4B,S4D).

For the TA and model groups (TA VS Model), the Volcano plot revealed 2,173 DEGs between them (Figure 5C), which were largely involved in several kinds of metabolic process, oxidation-reduction process, biosynthetic process, et al. (Figure 5D). The KEGG analysis predicted that these DEGs were mainly enriched in the metabolic pathways (especially the glycerophospholipid metabolism), PI3K/Akt signaling pathway, ECM production, and Steroid biosynthesis, et al. (Figures 5E,F).

### Effects of TA on Metabolism

The transcriptomics analysis predicted that the effect of TA on fibrogenesis might be associated with the regulation of the metabolism process. It is well known that metabolic disturbance causes excessive lipid accumulation, easily inducing fatty liver and fibrosis. Thus, the metabolism-related pathways were further analyzed.

As shown in Supplementary Figure S5, the PCA plot showed a relatively tight clustering among the QC samples; the Hotelling’s T2 Range revealed a high correlation coefficient, and the TIC (total ion chromatogram) diagram showed the same shape. The quality control analysis indicated that the method with high repeatability and stability could be used for the following assays.

**FIGURE 6:**
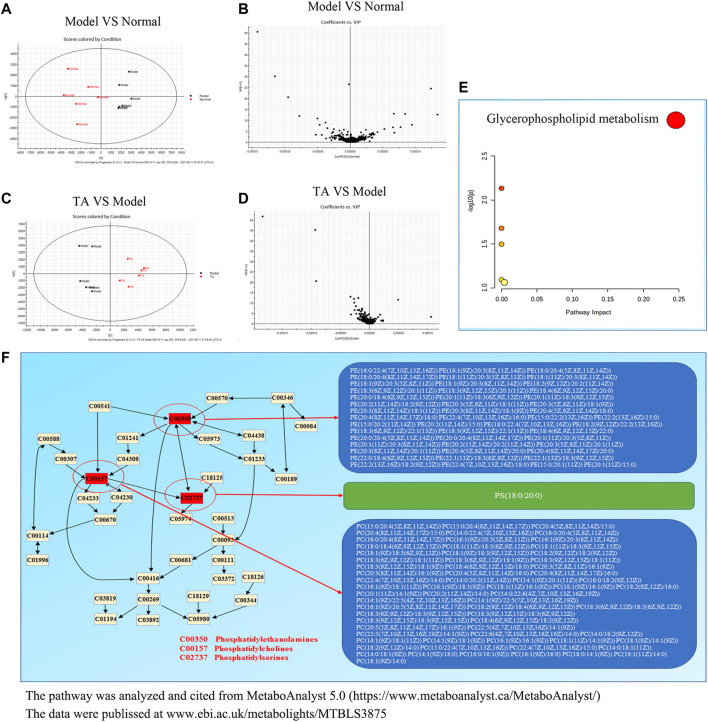
TA restored the metabolomic disorder induced by CCl4. **(A)**: Orthogonal partial least-square discrimination analysis (OPLS-DA) for Model VS Normal; **(B)**: The variable importance plot (VIP) diagram for Model VS Normal; **(C)**: OPLS-DA diagram for TA VS. Model; **(D)**: VIP diagram for TA VS Model; **(E)**: Effect of TA on the metabolomic pathways; **(F)**: The glycerophospholipid metabolism pathway.

The Orthogonal Partial Least-Square Discrimination Analysis (OPLS-DA) is a weighted average of the original scores, providing a good summary and displaying the separation between groups. As shown in Figure 6A, the OPLS-DA revealed an obvious separation between the model and normal control groups, which was confirmed by the variable importance plot (VIP) (Figure 6B). Further analysis indicated that CCl_4_ administration decreased 151 metabolites content and up-regulated 41 metabolites level, suggesting that CCl_4_ treatment led to significant metabolic disturbance. TA treatment might restore CCl_4_-induced metabolic change, as evidenced by the significant variance between the TA and model groups in the OPLS-DA and VIP diagram (Figures 6C,D). Importantly, six metabolic pathways were found by the analysis of MetaboAnalyst 5.0, including glycerophospholipid metabolism, sphingolipid metabolism, arachidonic acid metabolism, linoleic acid metabolism, alpha-linolenic acid metabolism and glycosylphosphatidylinositol-anchor biosynthesis (Supplemental Table S5 and Figure 6E). Among them, the glycerophospholipid metabolism pathway may be the most relevant (*p* < 0.05 and Impact >0.1). Further analysis indicated that TA might regulate the glycerophospholipid metabolism pathway by moderating the key metabolites, including phosphatidylserines, phosphatidylethanolamines and phosphatidylcholines (Figure 6F).

### Integrative Analysis of the Transcriptomics and Metabolomics Data

**FIGURE 7:**
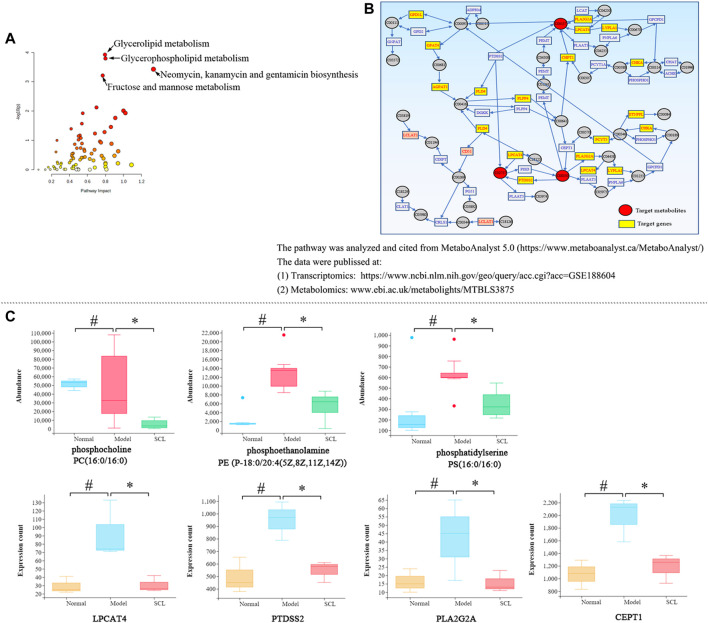
Integrated analysis of transcriptomics and metabolomics. **(A)**: The metabolic pathways; **(B)**: The glycerophospholipid metabolism pathway; **(C)**: The quantitative analysis of the key metabolites and DEGs. ^*^ *p* < 0.05 vs. the model control group. ^#^ *p* < 0.05 vs. the normal control group.

The integrative analysis of transcriptome and metabolome indicated that the DEGs and the metabolites were mainly clustered in six metabolic pathways (*p* < 0.05 and impact>0.5) (Supplemental Table S6 and Figure 7A). Among them, only the glycerophospholipid metabolism pathway included both the DEGs and the differential metabolites simultaneously; further analysis revealed that TA affected the synthesis of the key metabolites (phosphatidylserines, phosphatidylethanolamines and phosphatidylcholines) by regulating the genes expression of LPCAT4, PTDSS2, PLA2G2A and CEPT1 (Figure 7B). Also, the quantitative analysis confirmed that TA treatment decreased the content of phosphoethanolamine {PE [P-18:0/20:4 (5Z,8Z,11Z,14Z)]}, phosphocholine [PC (16:0/16:0)] and phosphatidylserine [PS (16:0/16:0)]; and decreased the expression of LPCAT4, PTDSS2, PLA2G2A and CEPT1 (Figure 7C).

### TA Inhibited the PI3K/Akt/mTOR Signaling Pathway

**FIGURE 8:**
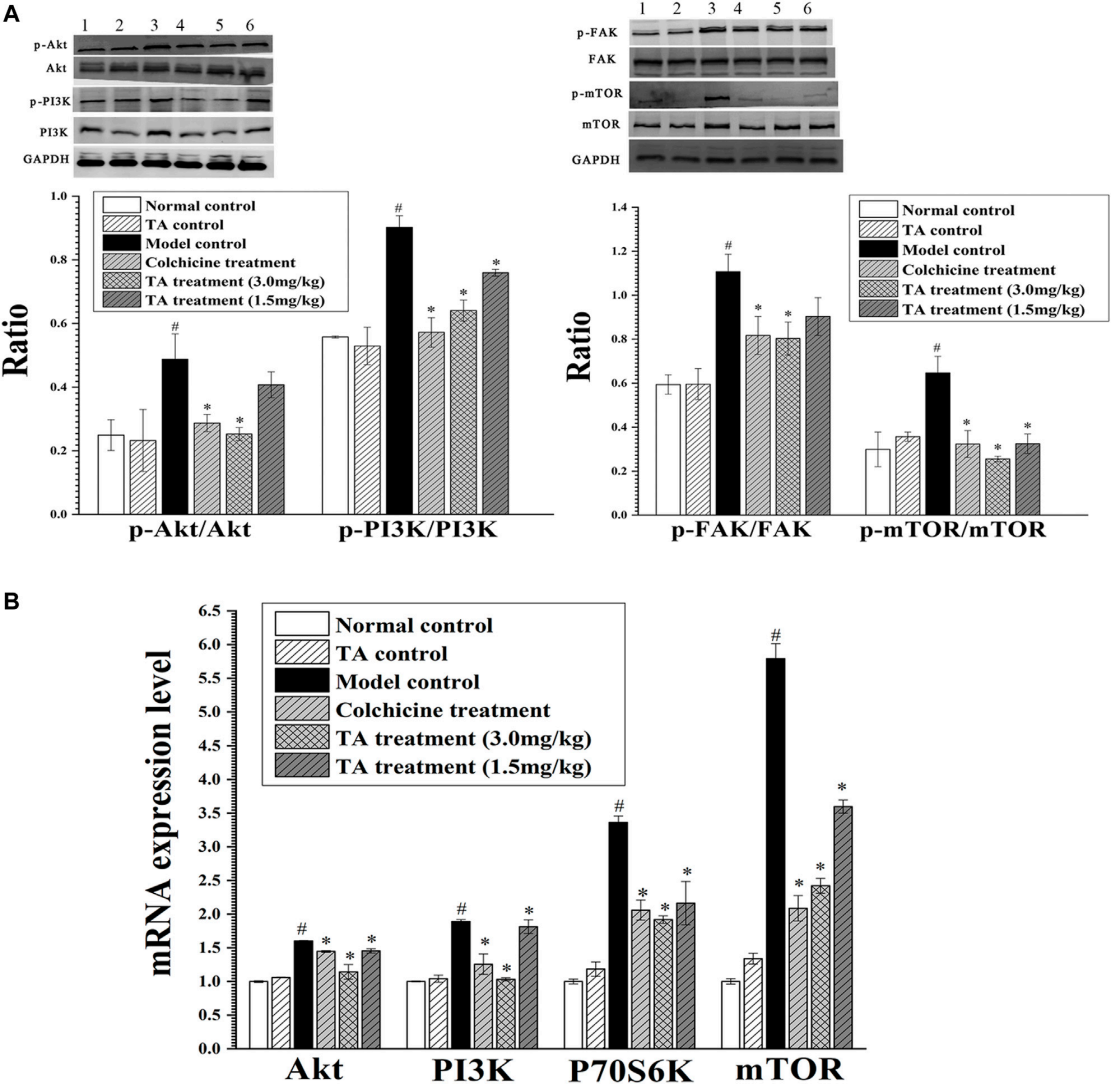
TA inhibited the PI3K/Akt/mTOR signaling pathway. **(A)** The phosphorylation of Akt, PI3K, FAK and mTOR protein was detected by Western blot analysis. Band one to six represented the normal control group, the TA control group, the model control group, the colchicine treatment group and the TA treatment groups (3.0 and 1.5 mg/kg), respectively. **(B)** The mRNA expression of Akt, PI3K, P70S6K and mTOR was detected by qPCR analysis. ^*^ *p* < 0.05 vs the model control group. ^#^ *p* < 0.05 vs the normal control group.

Based on the prediction of the transcriptomics analysis, the PI3K/Akt/mTOR signaling pathway was detected. As shown in Figure 8, CCl_4_ significantly increased the phosphorylation of Akt, PI3K, FAK and mTOR and the mRNA level of Akt, PI3K, P70S6K and mTOR compared with the normal control group (*p* < *0.05*). However, after treatment with TA, the expressions of these proteins and genes above were significantly decreased (*p < 0.05*), suggesting that TA could inhibit the PI3K/Akt/mTOR signaling pathway.

### TA Inhibited the NF-κB Signaling Pathway

**FIGURE 9:**
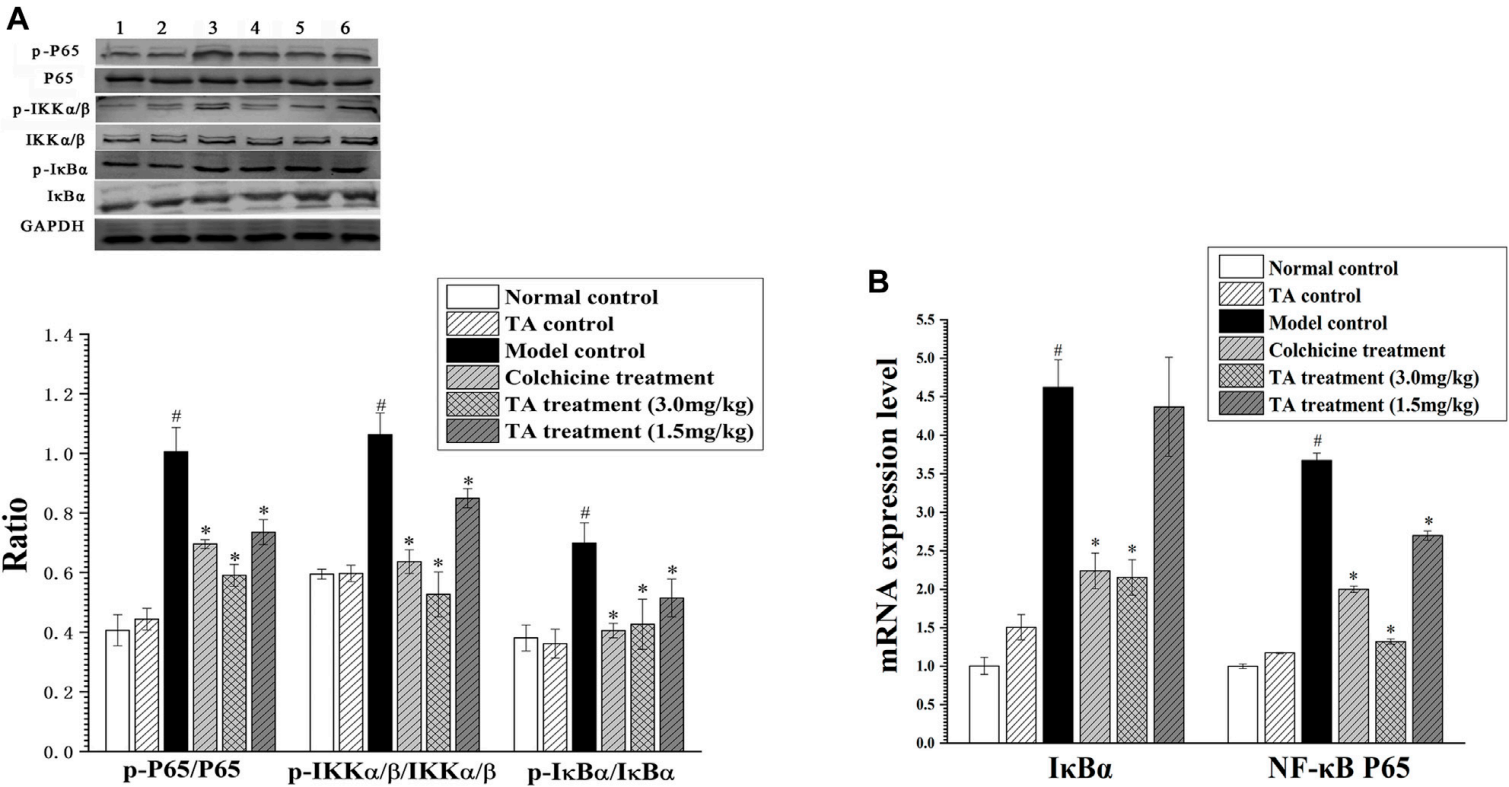
TA inhibited the NF-κB signaling pathway. **(A)** The key protein expression of the NF-κB signaling pathway was analyzed by Western blot analysis. Band one to six represented the normal control group, the TA control group, the model control group, the colchicine treatment group and the TA treatment groups (3.0 and 1.5 mg/kg), respectively. **(B)** The NF-κB p65 and IκBα mRNA expression was measured by qPCR analysis. ^*^ *p* < 0.05 vs. the model control group. ^#^ *p* < 0.05 vs. the normal control group.

The activation of the NF-κB pathway can initiate massive inflammatory cytokines secretion, aggravating hepatocyte necrosis. The results showed that CCl_4_ exposure significantly enhanced the phosphorylation of p65, IKKα/β and IκBα; while TA treatment decreased the phosphorylation of these proteins. Similarly, CCl_4_ increased the mRNA level of IκBα and NF-κB p65, but TA treatment reduced their mRNA levels (Figure 9). These data demonstrated that TA could inhibit the NF-κB signaling pathway and, as a result, alleviate inflammatory lesions.

## Discussion

The pathological examination showed that administration with CCl_4_ caused many infiltrations of inflammatory cells and destruction of liver lobules. However, TA treatment remarkably alleviated these symptoms. Besides, TA significantly restored the hepatic function indicators (ALT, AST, TBIL, and ALB) nearly to the normal level. In addition, the TUNEL staining revealed that TA treatment markedly decreased the positive apoptotic cells by regulating the expression of Bcl-2 and caspase families. Importantly, the Sirius staining revealed that TA treatment markedly reduced collagen deposition; also, TA treatment could decrease the content of Hyp, HA, and LN, as well as the expression of HPC III and col-IV. The data above indicated that TA could significantly alleviate CCl_4_-induced liver injury and fibrogenesis.

Transcriptomics analysis can reflect the global change of genes, which is a promising technique to elucidate complex pathogenic mechanisms. In this study, transcriptomic analysis was carried out to predict the potential targets of TA on liver fibrosis. The analysis indicated that there were 2,173 differentially expressed genes (DEGs) between the TA and model groups, and these DEGs could be functionally annotated in the liver fibrosis-related pathways, including the metabolic pathways (especially the glycerophospholipid metabolism), PI3K/Akt signaling pathway, ECM formation, and NF-κB signaling pathway.

Based on the prediction of transcriptomics, the effects of TA on metabolic profile and the relevant pathways were investigated. The OPLS-DA analysis showed that TA treatment resulted in 211 down-regulated and 131 up-regulated metabolites. These differential metabolites were mainly enriched in four metabolic pathways; among them, the glycerophospholipid metabolism pathway was the most relevant one (*p* < 0.05 and Impact >0.1). Further analysis indicated that the targets of TA to regulate the glycerophospholipid metabolism pathway included phosphatidylserines, phosphatidylethanolamines and phosphatidylcholines. It has been confirmed that phosphoethanolamines (PEs) and phosphocholines (PCs) are the key phospholipids of cell membranes (Chang et al., 2017), which are involved in the synthesis of the characteristic bilayer structure of cells. CCl_4_ exposure can strongly induce oxidative damage due to excessive free radicals (ROS), resulting in the destruction of cell membrane structure. Also, CCl_4_ treatment easily causes the disorder of PEs and PCs metabolism in rats with hepatic fibosis (Zhang et al., 2021). In this study, the contents of phosphoethanolamine {PE [P-18:0/20:4 (5Z,8Z,11Z,14Z)]}, phosphocholine (PC(16:0/16:0)) and phosphatidylserine [PS(16:0/16:0)] in the CCl_4_ model group were significantly increased, indicating an altered membrane phospholipids metabolism and the damage to the cell membrane; however, TA treatment significantly reversed these changes. Additionally, the integrative analysis of the transcriptome and metabolome revealed that TA could regulate the genes expression of LPCAT4, PTDSS2, PLA2G2A and CEPT1 and then inhibit the biosynthesis of phosphatidylserines, phosphatidylethanolamines and phosphatidylcholines. Our finding suggested that TA could restore the abnormal glycerophospholipid metabolism, which might be contributed to alleviating hepatocyte damage and fibrosis.

As the prediction of the transcriptomic analysis, the PI3K/Akt might also be involved in the regulation of TA on fibrogenesis. The PI3K/Akt/mTOR pathway is one of the critical signaling pathways, which can regulate cell proliferation and apoptosis (Wang et al., 2015). When stimulated, the PI3K is activated, turning into PI3K phosphorylated phosphatidylinositol 3, 4-triphosphate (PIP3). PIP3 promotes Akt phosphorylation, which regulates multiple signal transduction related to apoptosis. Phosphorylated mTOR awakens the downstream protein P70S6K to promote mRNA coding, translation and transcription, regulating cell growth and proliferation (Li et al., 2015; Dong et al., 2019). In this study, TA treatment significantly inhibited the expression of *α*-SMA and TGF-*β*, suggesting that TA could suppress HSCs activation and proliferation. Importantly, CCl_4_ exposure markedly activated the PI3K/Akt pathway in the fibrotic liver tissue, as evidenced by the significant increase in the phosphorylation of p-PI3K, p-Akt, p-mTOR and p-FAK. While treatment with TA significantly inhibited the phosphorylation levels of these proteins. Similarly, the results of RT-qPCR also showed that TA significantly inhibited the expression of the key genes of the PI3K/Akt/mTOR signaling pathway. These results suggested that TA inhibited HSCs activation by suppressing the PI3K/Akt/mTOR signaling pathway, which was vital for alleviating ECM accumulation and fibrogenesis.

In addition to the PI3K/Akt/mTOR signaling pathway, the NF-κB pathway might also be involved in the underlying mechanism of TA against liver fibrosis. NF-κB is a critical transcription factor in the inflammatory response, participating in the inflammatory response during liver fibrosis (Lu and Schwabe, 2011). Usually, IκBα binds to NF-κB to keep NF-κB a resting state in the cytoplasm. When hepatocytes are stimulated, IKK is activated and phosphorylated, and then promotes IκB phosphorylation. Thus, the IκBα-NF-κB compound is dissociated, and the NF-κB is exposed and activated, transferring into the nucleus. NF-κB activation can produce the inflammatory mediator genes and promote HSC activation and proliferation (Chen et al., 2019; Hu et al., 2020). The present study showed that TA treatment significantly increased the activity of SOD, GSH-Px and GSH-Rd, but decreased the content of MDA, suggesting that TA alleviated liver injury by inhibiting lipid peroxidation and effectively recruiting the anti-oxidative defense system. Besides, TA treatment significantly reduced the contents of TNF-α, IL-6 and MCP-1, while increased the level of IL-10 (an anti-inflammatory cytokine). These data suggested that the protective effect of TA on liver injury might be attributed to its anti-inflammatory ability. Interestingly, TA treatment significantly inhibited the phosphorylation of p-P65, p-IKK*α*/*β*, and p-IκB*α*. Similarly, the RT-qPCR results showed that TA significantly reduced the mRNA level of IκBα and NF-κB p65. These data indicated that TA ameliorated inflammatory injury by inhibiting the NF-κB signaling pathway.

## Conclusion

**FIGURE 10:**
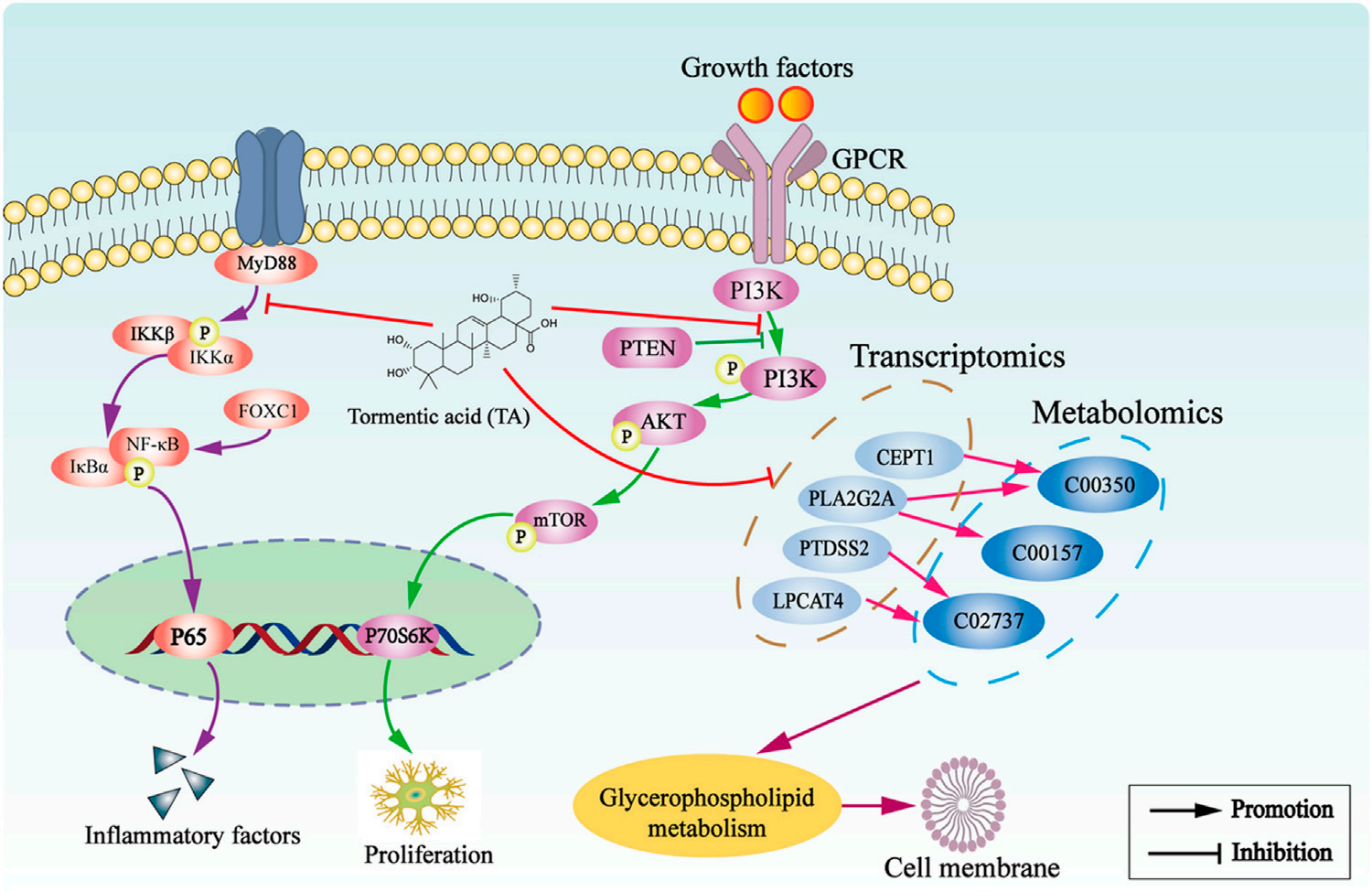
The underlying mechanism of TA against hepatic fibrosis.

TA treatment significantly ameliorates CCl_4_-induced liver fibrosis, which may be attributed to the inhibition of the glycerophospholipid metabolism pathway and the PI3K/Akt/mTOR and NF-κB signaling pathways (Figure 10).

## Data Availability

The datasets presented in this study can be found in online repositories. The names of the repository/repositories and accession number(s) can be found below: https://www.ncbi.nlm.nih.gov/geo/query/acc.cgi?acc= GSE188604; www.ebi.ac.uk/metabolights/MTBLS3875.
