# Supplementary material for: Tormentic Acid Ameliorates Hepatic Fibrosis in vivo by Inhibiting Glycerophospholipids Metabolism and PI3K/Akt/mTOR and NF-κB Pathways: Based on Transcriptomics and Metabolomics
Source: Front Pharmacol. 2022 Mar 11;13:801982. doi: 10.3389/fphar.2022.801982 (PMC8963336; doi:10.3389/fphar.2022.801982)
Supplement: Supplementary file 1 [file DataSheet2.pdf]

Raw data of TA anti-fibrosis's assays

Fig.1A H&E staining (Olympus CX33 with ImageView software, 400×)

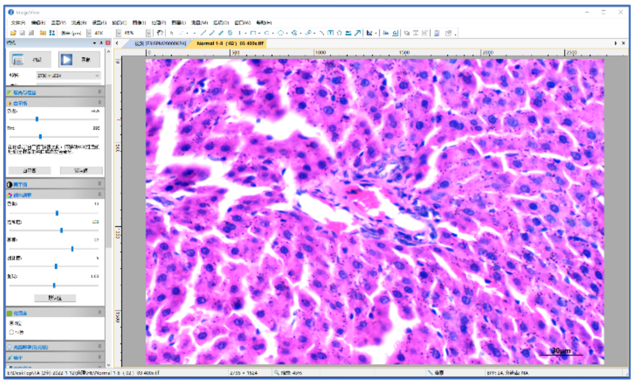

Normal group

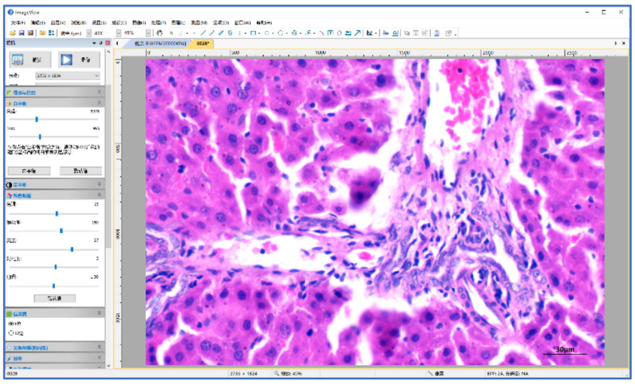

TA control group

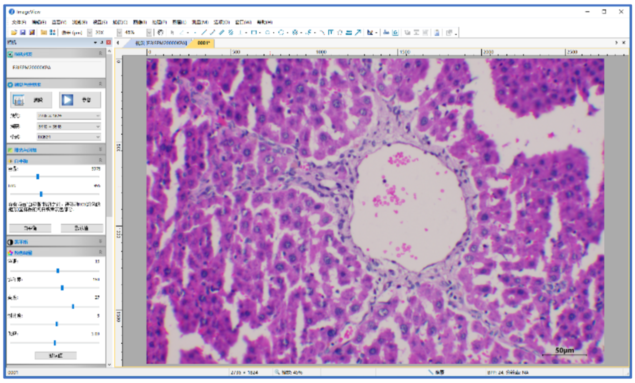

Model group

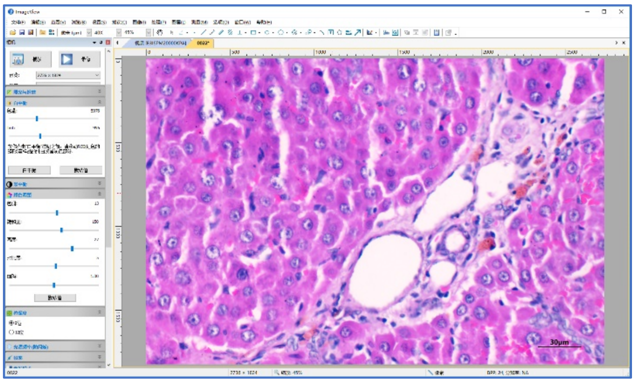

Colchicine group

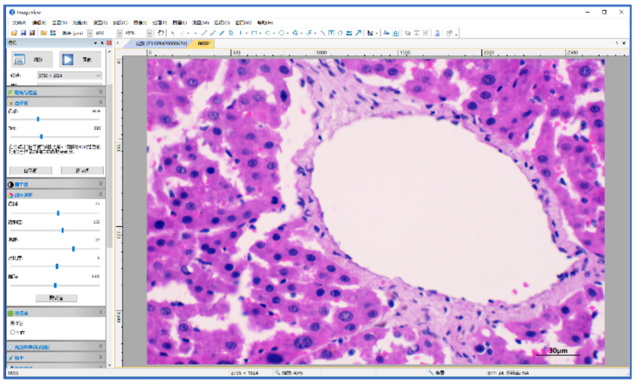

TA (high dose 3mg/kg)

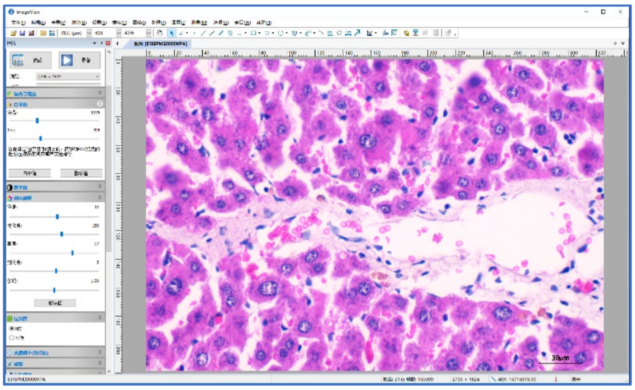

TA (low dose 1.5mg/kg)

Fig.2A Sirius staining (Olympus CX33 with ImageView software, 100×)

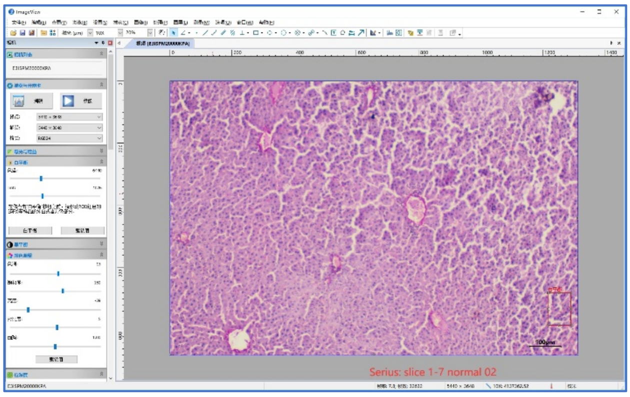

Normal group

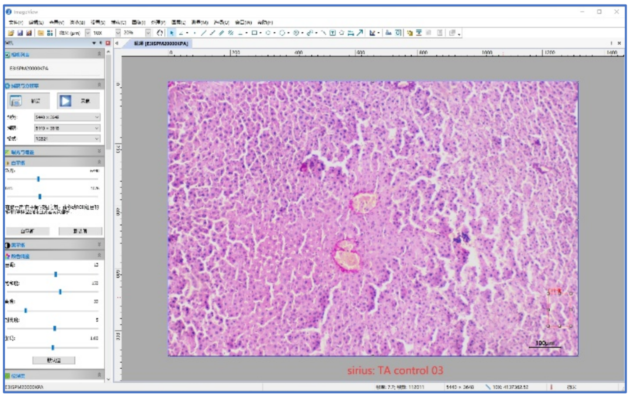

TA control group

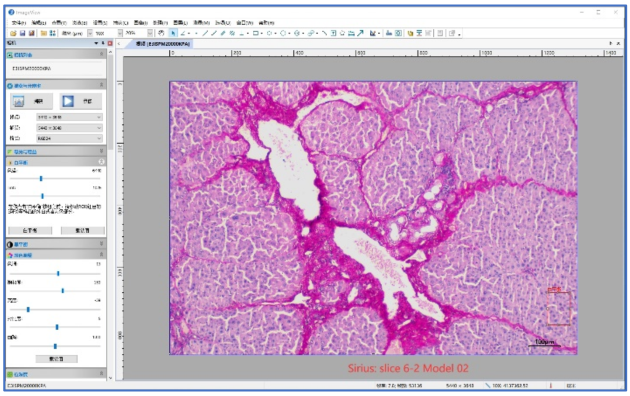

Model group

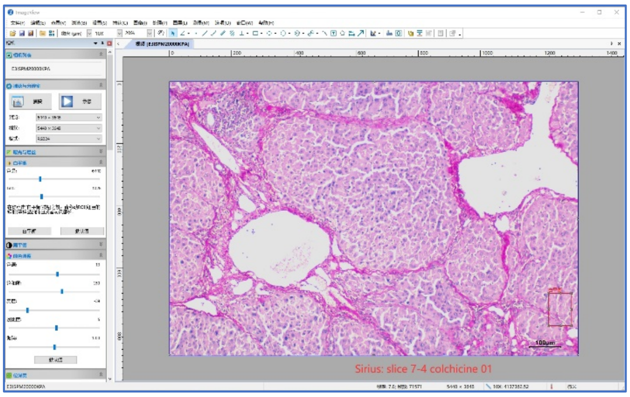

Colchicine group

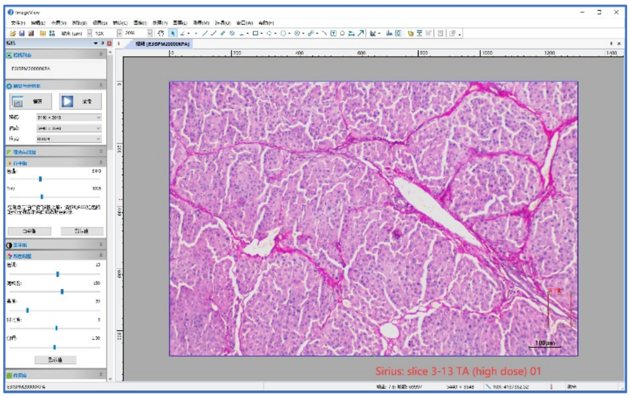

TA (high dose 3mg/kg)

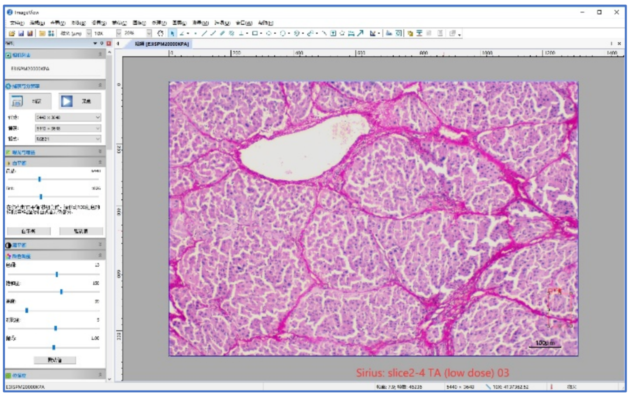

TA (low dose 1.5mg/kg)

Fig. 3A TUNUL staining (Olympus CX33 with ImageView software, 100×)

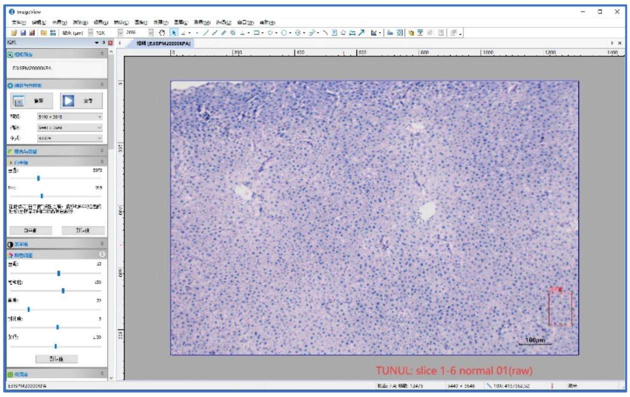

Normal group

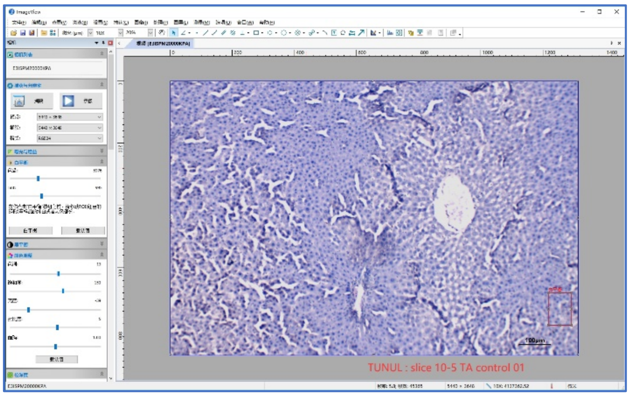

TA control group

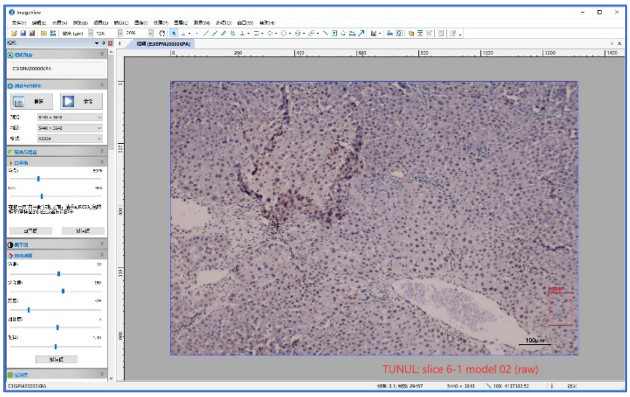

Model group

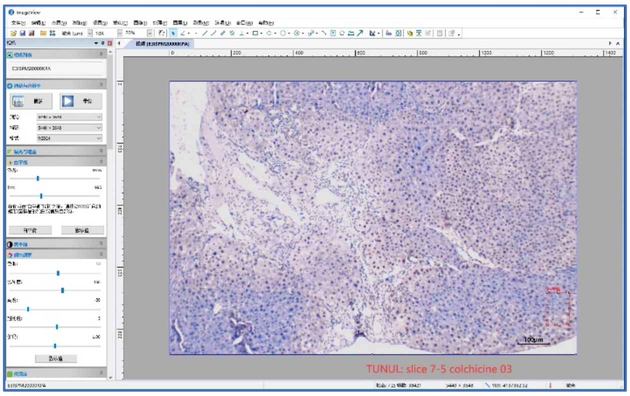

Colchicine group

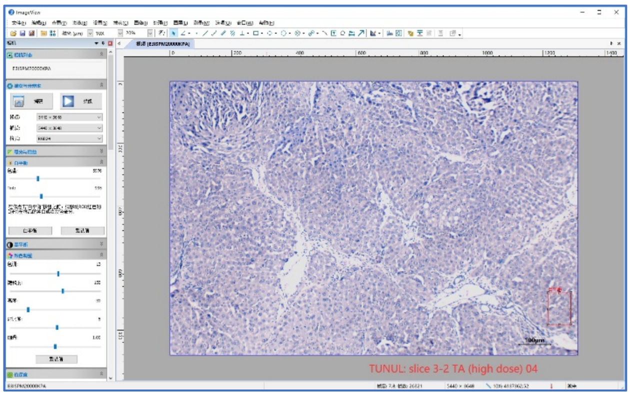

TA (high dose 3mg/kg)

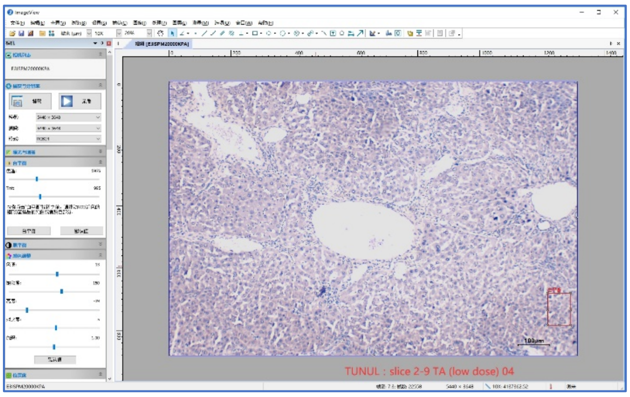

TA (low dose 1.5mg/kg)

The raw bands of Western blot

Fig. 2C α-SMA, Col-I and Col-III

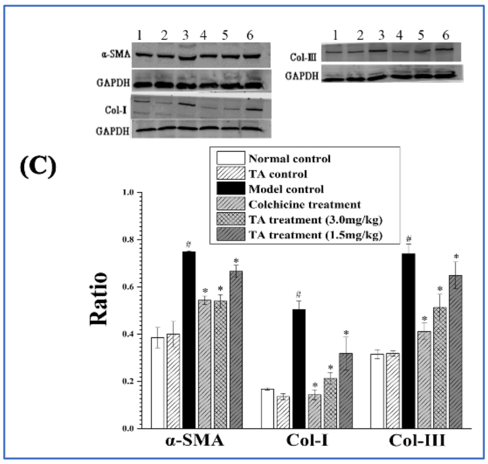

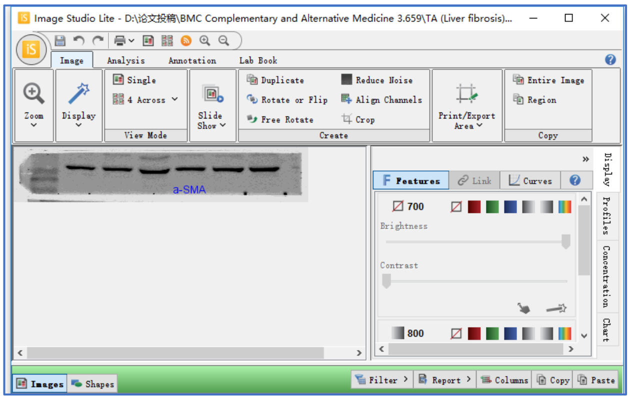

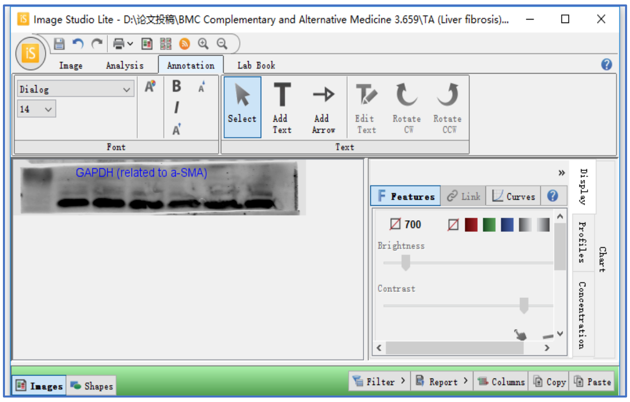

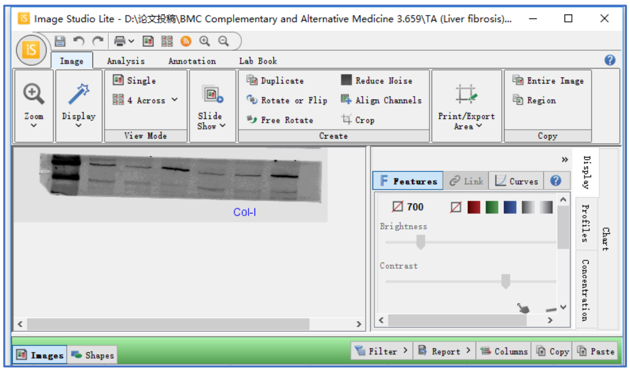

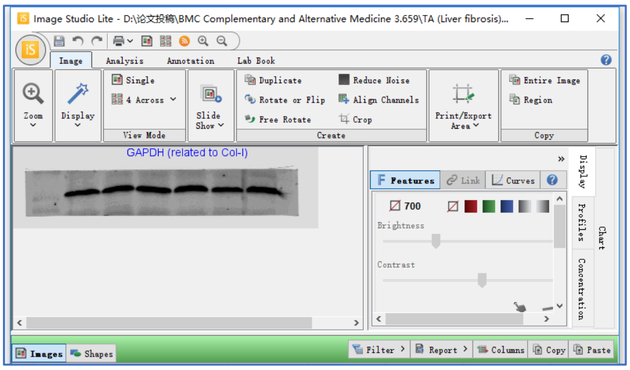

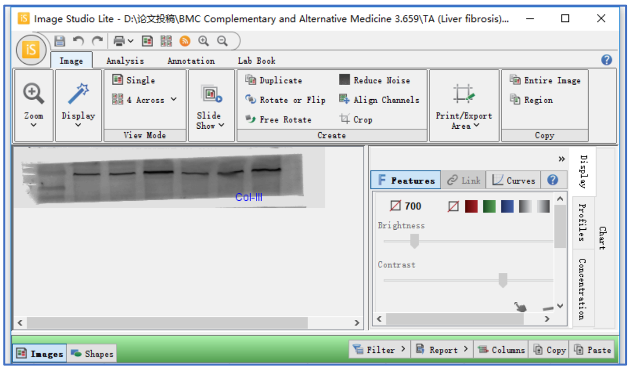

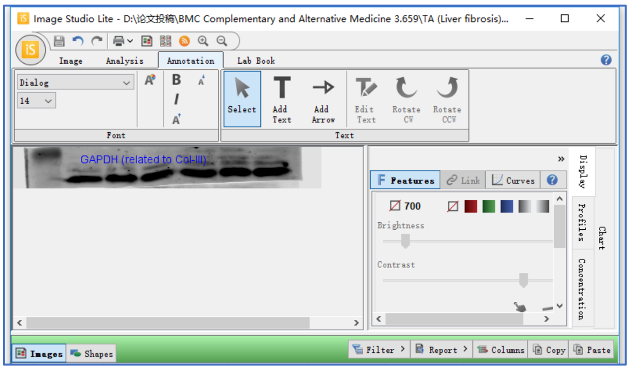

Fig. 3. TA decreased hepatocyte apoptosis

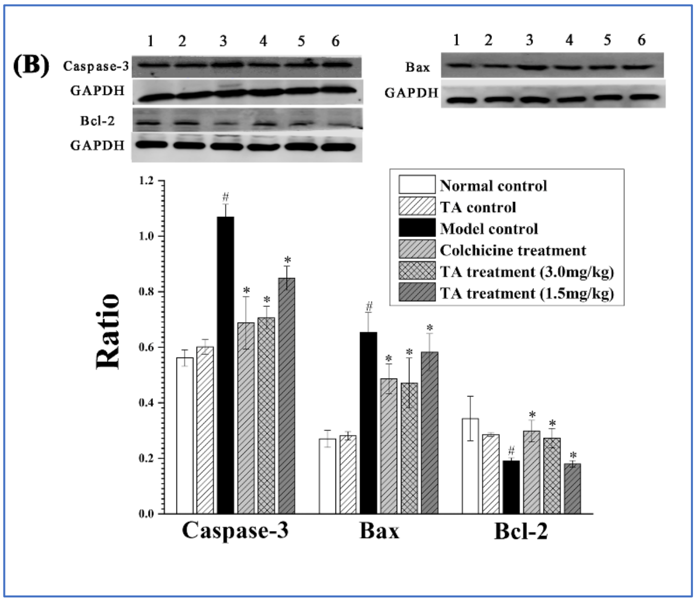

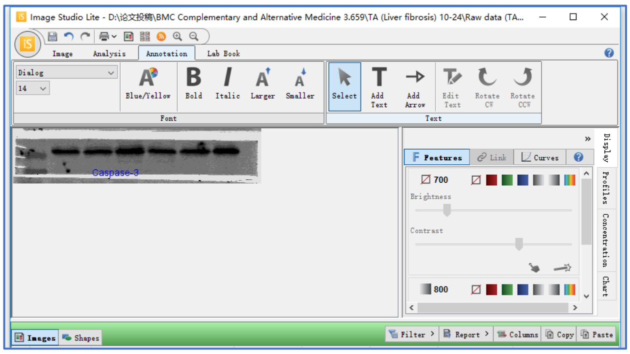

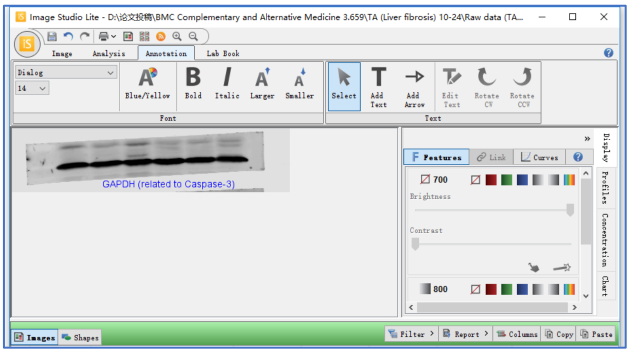

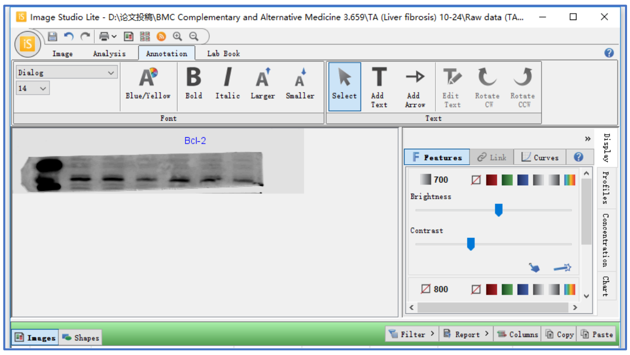

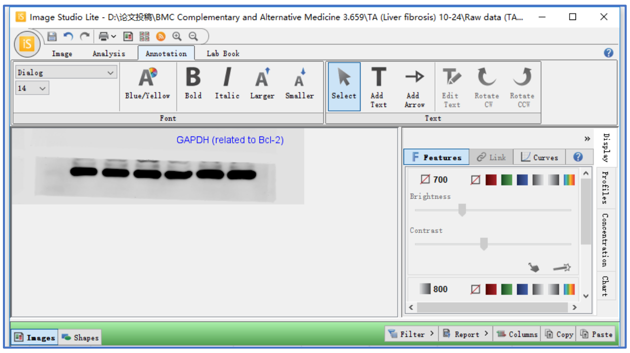

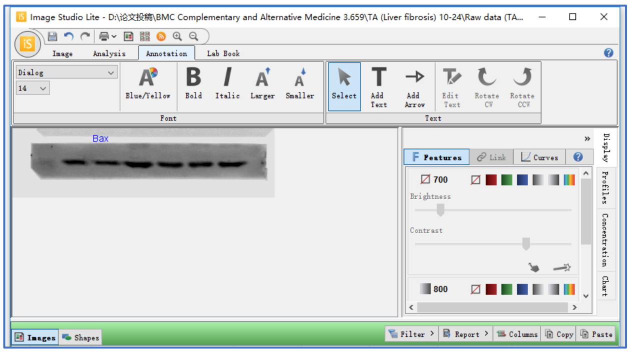

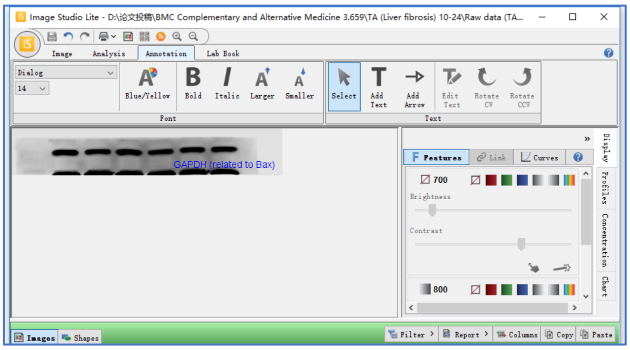

Fig. 8. TA inhibited the PI3K/Akt/mTOR signaling pathway

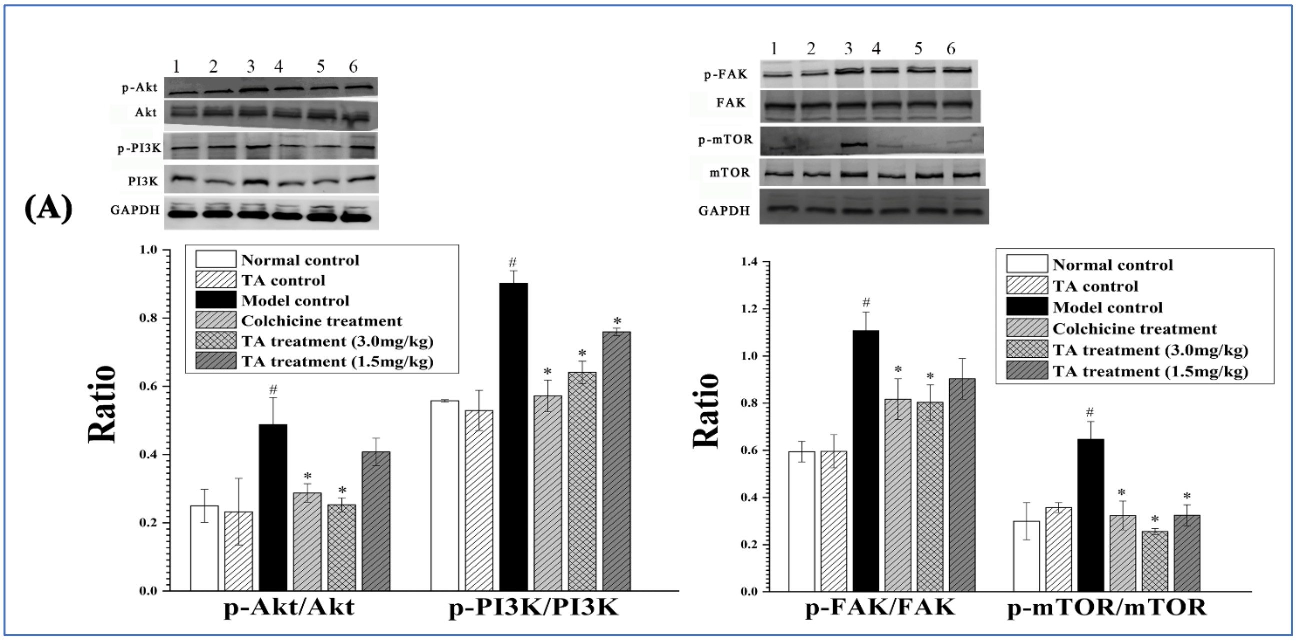

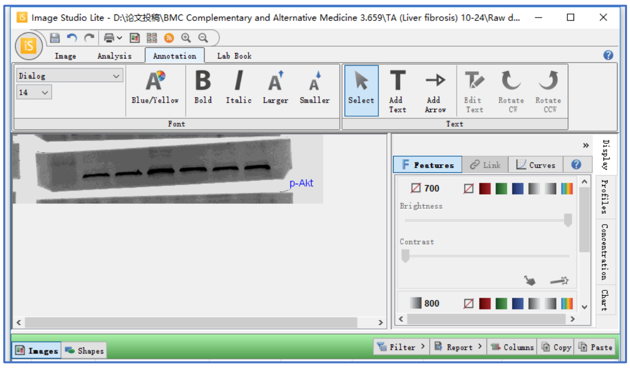

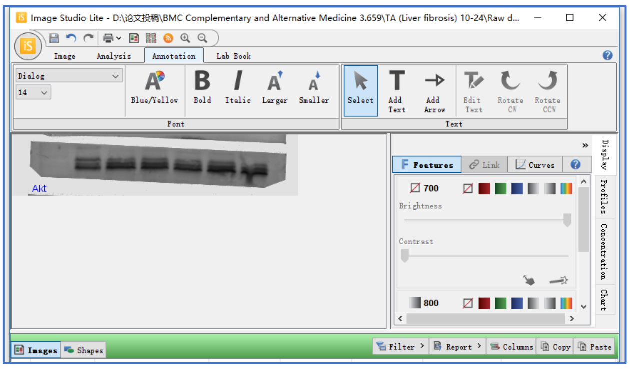

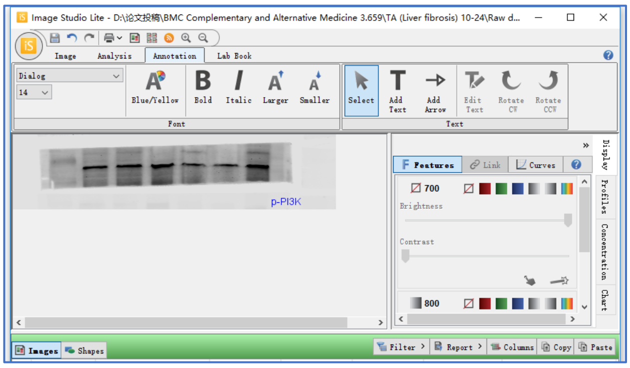

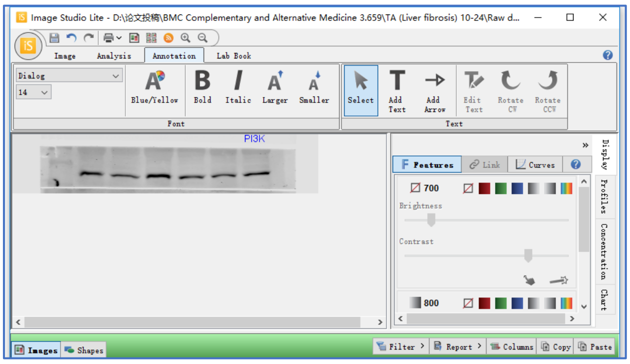

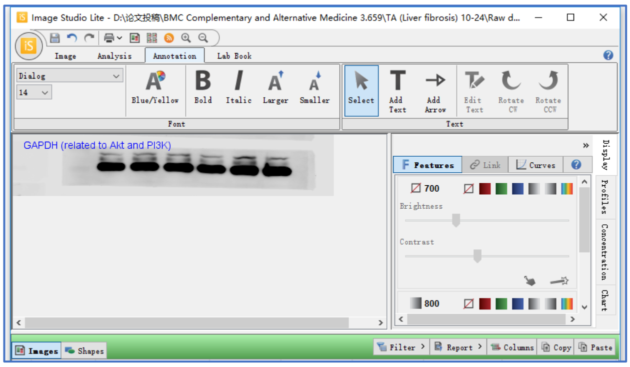

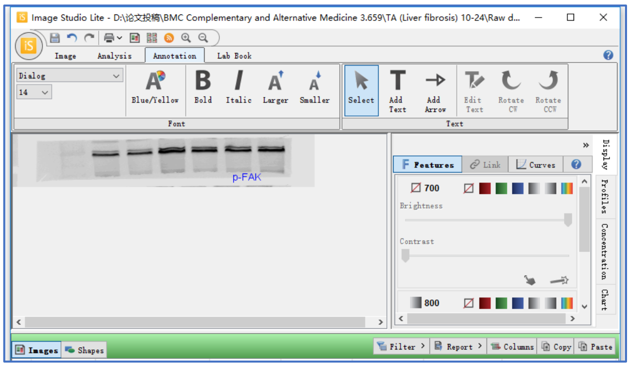
Image Studio Lite - D:\论文投稿\BMC Complementary and Alternative Medicine 3.659\TA (Liver fibrosis) 10-24\Raw d...
p-FAK

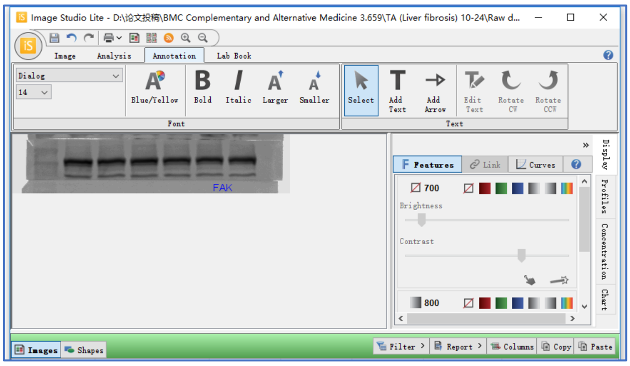
Image Studio Lite - D:\论文投稿\BMC Complementary and Alternative Medicine 3.659\TA (Liver fibrosis) 10-24\Raw d...
FAK

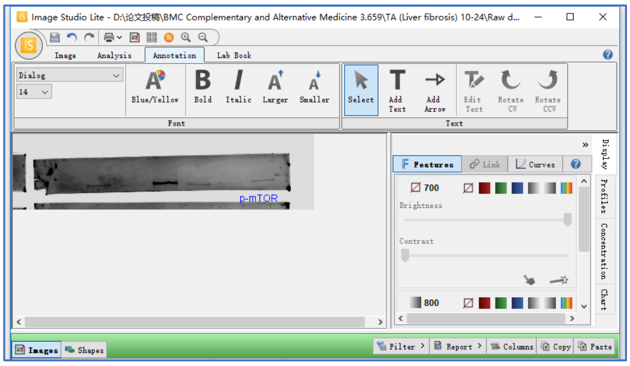
Image Studio Lite - D:\论文投稿\BMC Complementary and Alternative Medicine 3.659\TA (Liver fibrosis) 10-24\Raw d...
p-mTOR

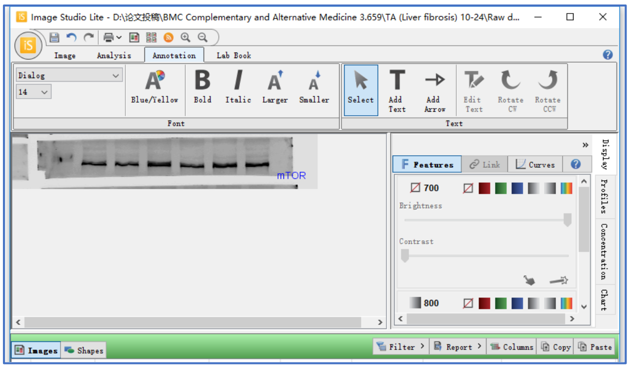
Image Studio Lite - D:\论文投稿\BMC Complementary and Alternative Medicine 3.659\TA (Liver fibrosis) 10-24\Raw d...
mTOR

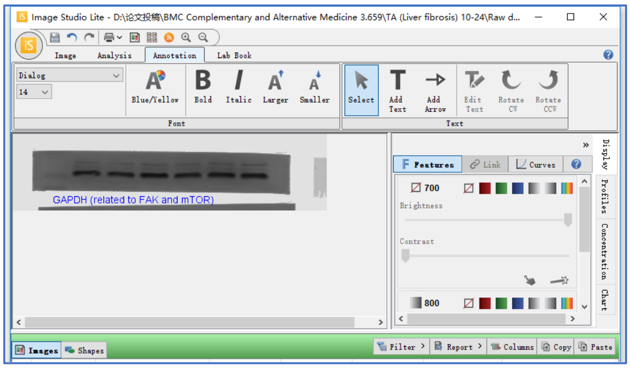
Image Studio Lite - D:\论文投稿\BMC Complementary and Alternative Medicine 3.659\TA (Liver fibrosis) 10-24\Raw d...
GAPDH (related to FAK and mTOR)

Fig. 9. TA inhibited the NF-κB signaling pathway

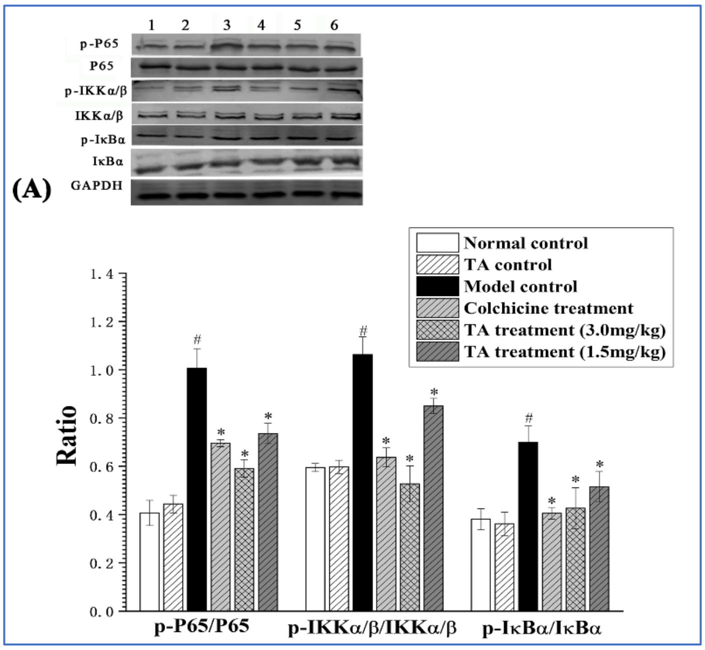

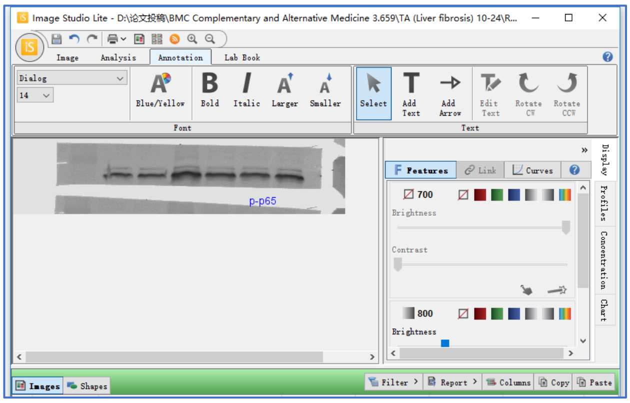

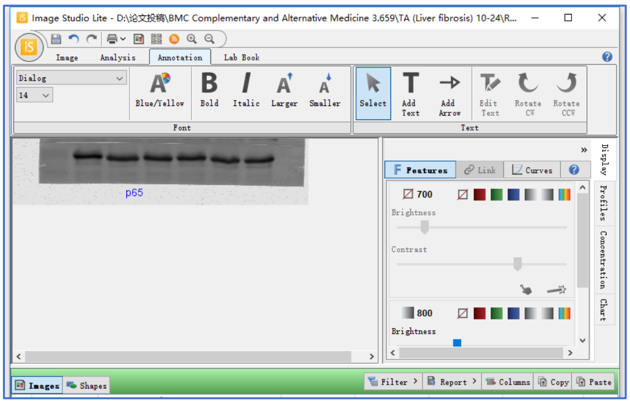

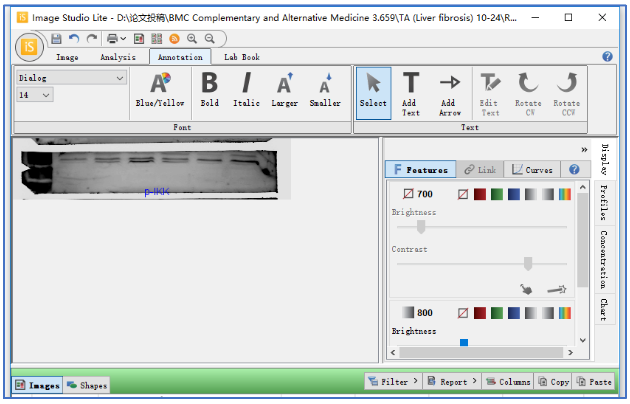

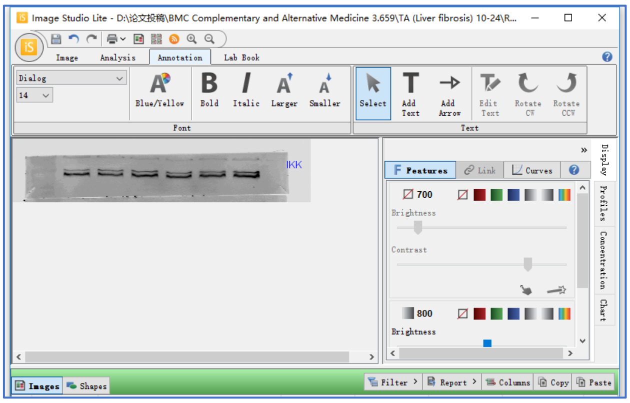

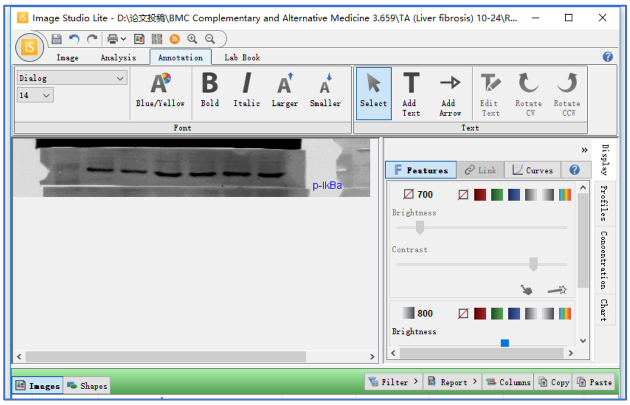
p-IkBa

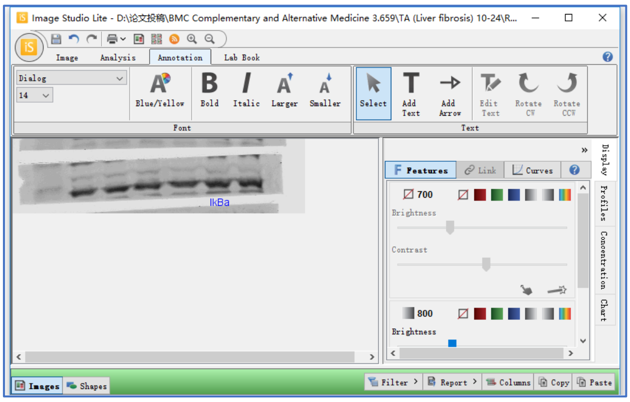
IkBa

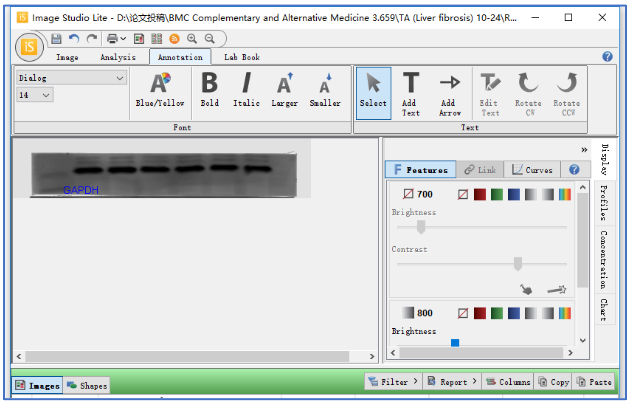
GAPDH
