# Supplementary material for: Tormentic Acid Ameliorates Hepatic Fibrosis in vivo by Inhibiting Glycerophospholipids Metabolism and PI3K/Akt/mTOR and NF-κB Pathways: Based on Transcriptomics and Metabolomics
Source: Front Pharmacol. 2022 Mar 11;13:801982. doi: 10.3389/fphar.2022.801982 (PMC8963336; doi:10.3389/fphar.2022.801982)
Supplement: Supplementary file 2 [file DataSheet1.pdf]

Supplemental materials

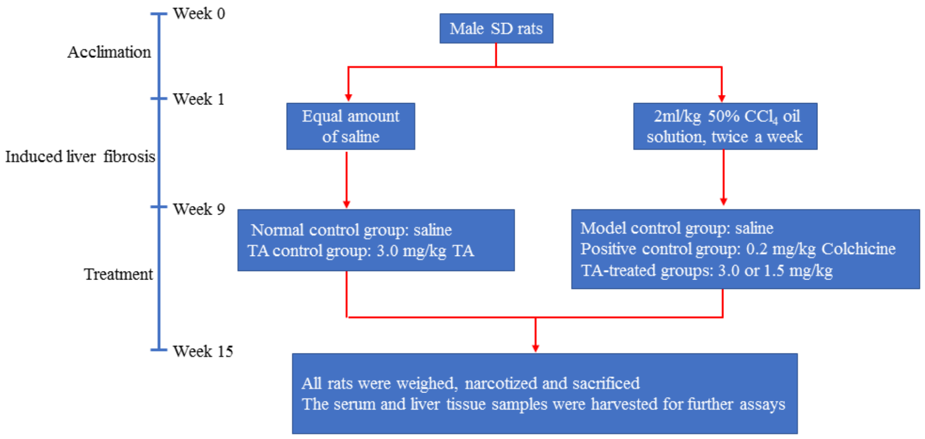

Fig. S1 The experiment schedule

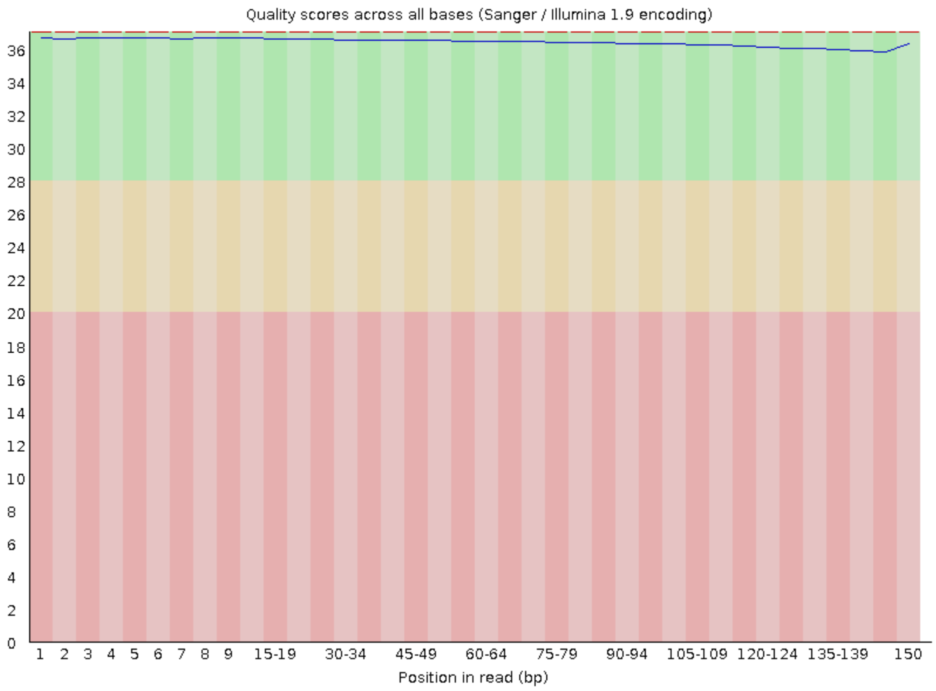

Fig. S2 Quality scores across all bases

X-axis: the position in read; Y-axis: the value of base quality. The Red line means the median; the blue line represents the average value; the yellow line represents the 25%-75% range.

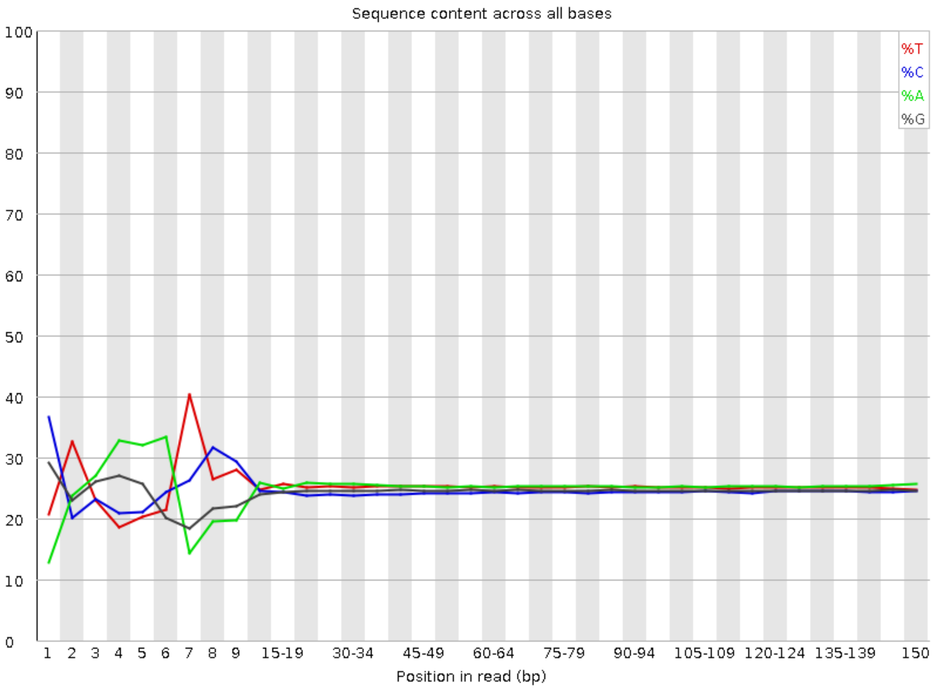

Fig. S3 Sequence content across all bases

X-axis: the position in read; Y-axis: the ratio of base.

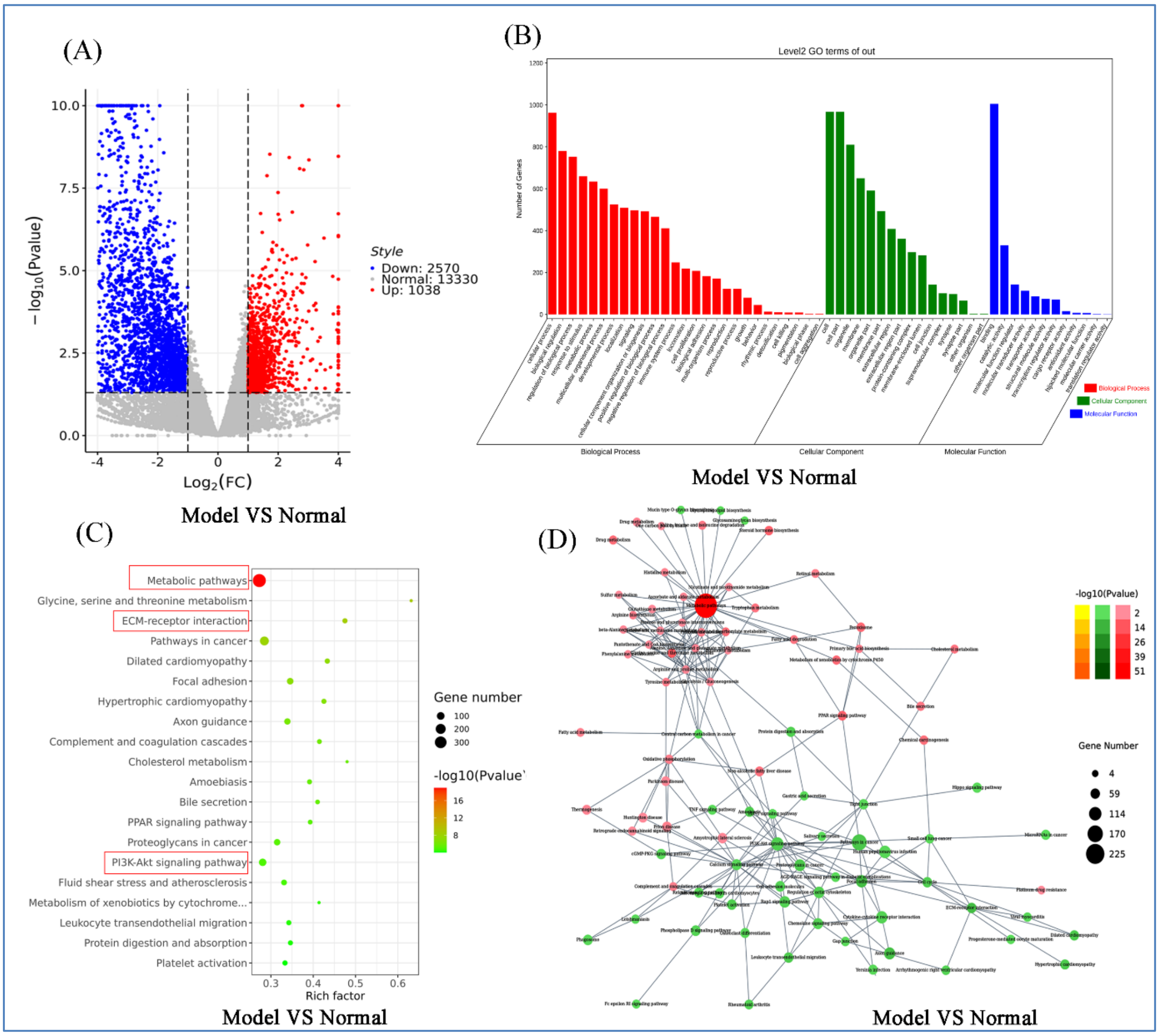

Fig. S4 Transcriptomics analysis for Model VS Normal

A: Venn diagram; B: Heatmap; C: Volcano plot; D: Gene ontology (GO) analysis; E: KEGG pathway analysis; F: Network was built based on the relationship between KEGG Pathways.

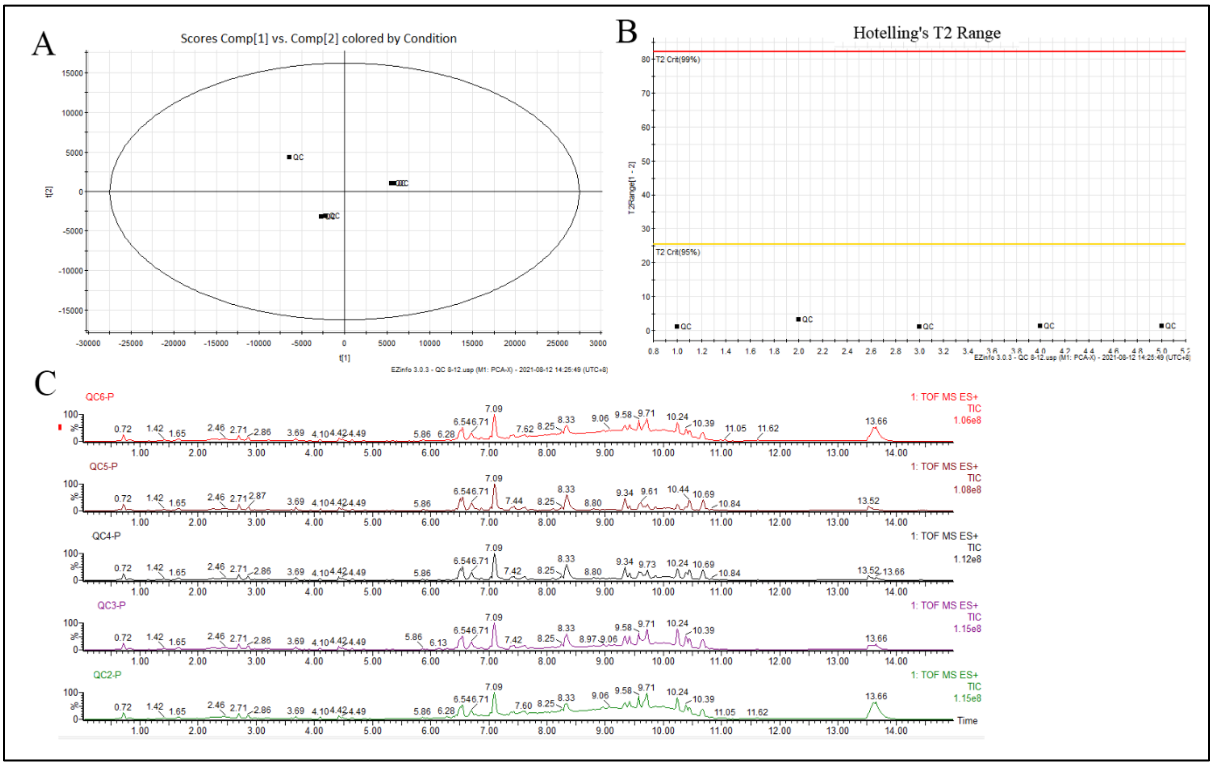

Fig. S5 Quality control (QC) samples

A: PCA (Principal Component Analysis) Score Plot; B: Hotelling's T2 Range; C: Typical total ion chromatograms (TICs).

Table S1 The gradient elution for UPLC-ESI-QTOF MS analysis

Time (min)	Flow (ml/min)	% A	% B	Curve
Initial	0.400	95.0	5.0	6
1.0	0.400	80.0	20.0	6
2.5	0.400	60.0	40.0	6
9.0	0.400	10.0	90.0	6
10.0	0.400	0.0	100.0	6
12.5	0.400	0.0	100.0	6
14.0	0.400	95.0	5.0	6
15.0	0.400	95.0	5.0	6

A: HPLC-grade water containing 0.1 % formic acid; B: HPLC-grade acetonitrile containing 0.1 % formic acid.

Table S2 The sequences primers for qPCR

Genes	Sense primer (5'-3')	Anti-sense primer (5'-3')
GAPDH	GGCACAGTCAAGGCTGAGAATG	ATGGTGGTGAAGACGCCAGTA
Col-I	GACATGTTCAGCTTTGTGGACCTC	AGGGACCCTTAGGCCATTGTGTA
Col-III	TTTGGCACAGCAGTCCAATGTA	GACAGATCCCGAGTCGCAGA
TGF-β	CATTGCTGTCCCGTGCAGA	AGGTAACGCCAGGAATTGTTGCTA
Bax	GACATGTTTTCTGACGGCAA	CCCAAAGTAGGAGAGGAGGC
Bcl-2	CACCCCTGGCATCTTCTCCT	GTTGACGCTCCCCACACACA
PI3K	GCATCAGTGGCTCAAGGACAAG	CAAGATAAAGGTTGCCACGCAGTA
Akt	ATGGACTTCCGGTCAGGTTCA	GCCCTTGCCCAGTAGCTTCA
mTOR	GCTTATCAAGCAAGCGACATCTCA	TCCACTGGAAGCACAGACCAAG
P70S6K	AAATCCGATCGCCTCGAAGA	CACTTGTTTCCATTGGGTATTCCAC
IκBα	TGACCATGGAAGTGATTGGTCAG	GATCACAGCCAAGTGGAGTGGA
NF-κB P65	CGACGTATTGCTGTGCCTTC	TTGAGATCTGCCCAGGTGGTA

Table S3 The statistical result of raw reads and clean reads

Sample Name	Raw Reads	Clean Reads	Raw Bases (G)	Clean Bases (G)	Q20 (%)	Q30 (%)	GC Content (%)
Normal 1	44481548	38418206	6.67	5.76	98.87	95.63	49.22
Normal 2	56509214	49703434	8.48	7.46	98.94	95.79	49.26
Normal 3	46889182	40135840	7.03	6.02	98.85	95.55	48.90
Model 1	41893536	35826064	6.28	5.37	98.66	94.99	50.43
Model 2	41844660	35713216	6.28	5.36	98.74	95.20	48.45
Model 3	41961620	36095164	6.29	5.41	98.68	95.02	48.68
TA 1	43349624	37480058	6.50	5.62	98.75	95.23	49.43
TA 2	39919276	34667654	5.99	5.20	98.69	95.05	49.24
TA 3	46049212	39288328	6.91	5.89	98.79	95.38	48.70

Raw reads: the raw reads numbers; Clean reads: the clean reads numbers; Raw bases (G): the bases number of raw reads (the unit is G); Clean bases (G): the bases number of clean reads; Q20 (%): the reads number with the accuracy of bases sequencing > 99% / the total reads number; Q30 (%): the reads number with the accuracy of bases sequencing > 99.9% / the total reads number; GC Content (%): the GC bases/the total bases.

Table S4 Aligning clean reads to reference genome

Sample	Normal 1	Normal 2	Normal 3	Model 1	Model 2	Model 3	TA 1	TA 2	TA 3
Number of input reads	19209103	24851717	20067920	17913032	17856608	18047582	18740029	17333827	19644164
Average input read length	300	300	300	300	300	300	300	300	300
Uniquely mapped reads number	16675705	21586925	17300796	15678794	15858192	15991078	16629249	15324476	17213271
Uniquely mapped reads (%)	86.81%	86.86%	86.21%	87.53%	88.81%	88.61%	88.74%	88.41%	87.63%

Table S5 The metabolic pathways

Pathway	p	-log (p)	Holm p	Impact
Glycerophospholipid metabolism	0.0012358	2.9081	0.1038	0.24596
Sphingolipid metabolism	0.007372	2.1324	0.61188	0.00
Arachidonic acid metabolism	0.020999	1.6778	1.0	0.00
Linoleic acid metabolism	0.031885	1.4964	1.0	0.00
alpha-Linolenic acid metabolism	0.081002	1.0915	1.0	0.00
Glycosylphosphatidylinositol (GPI)-anchor biosynthesis	0.086981	1.0606	1.0	0.00399

Table S6 The pathways of the integrated analysis between transcripts and metabolites

Pathway	p	-log (p)	Holm p	Impact
Glycerolipid metabolism	1.1956E-4	3.9224	0.010043	0.79412
Glycerophospholipid metabolism	1.6139E-4	3.7921	0.013395	0.8
Neomycin, kanamycin and gentamicin biosynthesis	3.7572E-4	3.4251	0.030809	1.3333
Fructose and mannose metabolism	6.1472E-4	3.2113	0.012909	0.76923
Glycolysis or Gluconeogenesis	0.007568	2.121	0.60548	0.7
Citrate cycle (TCA cycle)	0.0098055	2.0085	0.77464	1.0
